# Scalable Crackle‐Lithography for Multifunctional Enclosed Plastic Nanofluidic Devices

**DOI:** 10.1002/advs.76980

**Published:** 2026-08-06

**Authors:** Tirumala Rao Dumpala, S. Kiruthika, Siddhi Vinayak Pandey, Junyang Zhang, Solleti Goutham, Marcos Vinicius Surmani Martins, Harry Whittingham, Boya Radha, Ashok Keerthi

**Affiliations:** ^1^ Department of Physics and Astronomy School of Natural Sciences The University of Manchester Oxford Road Manchester UK; ^2^ Department of Chemistry School of Natural Sciences The University of Manchester Oxford Road Manchester UK; ^3^ National Graphene Institute The University of Manchester Booth Street East Manchester UK; ^4^ Department of Physics School of Electrical and Electronics Engineering SASTRA Deemed to Be University Thanjavur Tamil Nadu India; ^5^ Photon Science Institute The University of Manchester Oxford Road Manchester UK

**Keywords:** confined chemical reactions, crackle‐lithography, fluorescence microscopy, high‐throughput fabrication, ionic memristor, nanochannels, nanofluidics, PETG

## Abstract

Nanofluidic devices have attracted considerable interest from both the scientific community and industry owing to their broad potential in molecular transport, sensing, energy conversion, and information technologies. However, the widespread adoption of nanofluidic devices has been hindered by fabrication methods that rely on expensive infrastructure, sophisticated nanofabrication facilities, and labor‐intensive processing. Here, we present crackle‐lithography, a simple, scalable, and cost‐effective approach for fabricating enclosed plastic nanofluidic devices. By harnessing spontaneous crack formation as a lithographic template, highly interconnected nanochannel networks are transferred into polyethylene terephthalate glycol (PETG) substrates through oxygen‐plasma etching and subsequently sealed by thermal bonding to produce robust nanofluidic devices. The fabrication strategy is cleanroom‐free, highly scalable, and compatible with high‐throughput manufacturing, making it attractive for practical implementation. The resulting devices are used for studying molecular transport of gases, water, and ions, exhibit history‐dependent ionic conduction with tunable memristive behavior and enable confined electroless copper deposition within the nanochannels. Their high optical transparency further allows real‐time fluorescence imaging of transport processes. Together, these results establish crackle‐lithography as a versatile and accessible platform for scalable nanofluidic device fabrication with potential applications in sensing, confined chemistry, neuromorphic ionics, and next‐generation nanofluidic technologies.

## Graphical abstract

Crackle‐lithography enables scalable fabrication of enclosed plastic‐substrate‐based nanofluidic devices with interconnected nanochannels that enable molecular transport, optical interrogation, confined electroless copper deposition, and ionic memristive behavior.

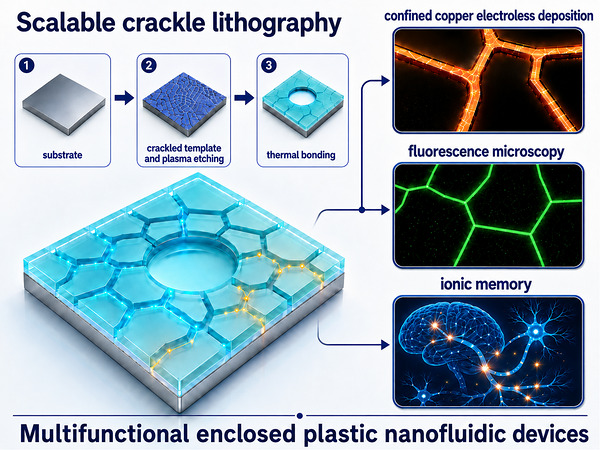

## 1 Introduction

Nanofluidics broadly refers to the study and control of fluids confined within conduits or channels with characteristic dimensions in the nanometer range, where interfacial and molecular interactions dominate over bulk fluid behavior. At these scales, conventional fluid dynamics no longer apply, and phenomena such as surface charge regulation, molecular sieving, and ion selectivity emerge. For example, unexpected and exotic properties like fast water flows in carbon channels [1, 2, 3], electrically dead water [4], and ballistic molecular transport [5] have been observed in the nanometer regime. One of the critical reasons behind these phenomena is confinement in channels with a high surface‐to‐volume ratio. Hence, nanochannels have gained much prominence in recent years owing to their extremely high potential for unveiling new physical phenomena. Although much progress has been made in recent years yet developing robust and scalable platforms for applications remains a major challenge, as most existing nanofluidic devices rely on complex fabrication processes and state‐of‐the‐art infrastructure.

Biological nanochannels, ubiquitous in nature, demonstrate how efficient nanometric transport can be achieved and offer clear cues for design. Aquaporins facilitate selective filtration and osmotic balance [6]; neuronal channels transduce extracellular signals into cellular responses [7]; and nanochannels in xylem tissues facilitate nutrient and water transport against gravity through capillary action [8]. The branching networks observed in leaf veins optimize resource distribution [9, 10], inspiring the development of analogous artificial patterns for advanced nanofluidic applications.

Conventional nanofabrication methods include techniques like electron beam lithography, reactive ion plasma etching processes, the deposition of thin films, etc. The most crucial part of nanochannel fabrication is patterning the channels, i.e., lithography. Currently, techniques like photolithography, electron beam lithography, focused ion beam lithography, nanoimprint lithography, and scanning probe lithography are most often used for this process. Making patterns at micro‐ or nanoscales has always been a technology‐intensive and labor‐intensive task, with the requirement of highly sophisticated infrastructure (instruments, facilities, and a highly skilled workforce) [11, 12, 13].

Crackle‐lithography [14] is a crack‐assisted fabrication technique primarily inspired by nature. We observe periodic patterns in nature ranging from mud cracking [15, 16] to the skin of crocodiles and elephants [17, 18]. Cracking on the surface is an unfavorable outcome in most fabrication processes, but patterned cracks can be used in various micro‐fabrication techniques with careful consideration. Recent advances have shown that high‐resolution and high‐throughput patterns can be obtained from induced cracks [19, 20]. These micrometer cracks have also been employed in various functional applications like flexible electronics (transparent conductive electrodes) [21, 22], optical devices [23, 24], supercapacitors [25, 26], liquid crystal devices [27], and sensors [28, 29, 30]. Previous studies have demonstrated the potential of crack‐assisted approaches for nanoscale pattern generation [31], crack‐derived nanochannels [32], and micro/nanofluidic diffusion control [33, 34]. These works provided important foundations for using crack formation as a low‐cost route to create nanoscale features. However, the integration of crack‐derived networks into enclosed nanofluidic devices capable of supporting confined chemical reactions, nanofluidic ionic transport, and ionic memristive behavior remains largely unexplored.

Here, we explore crackle‐lithography to generate nanoscale patterns on polyethylene terephthalate glycol (PETG) substrates, utilizing cracked masks as templates for selective etching of channels on the polymer/plastic substrate and subsequently use those substrates for fabricating nanofluidic devices. These devices will provide an affordable and scalable means to investigate nanoscale transport phenomena and unlock new opportunities in molecular sensing, energy conversion, and soft‐matter engineering.

## 2 Results and Discussion

**FIGURE 1:**
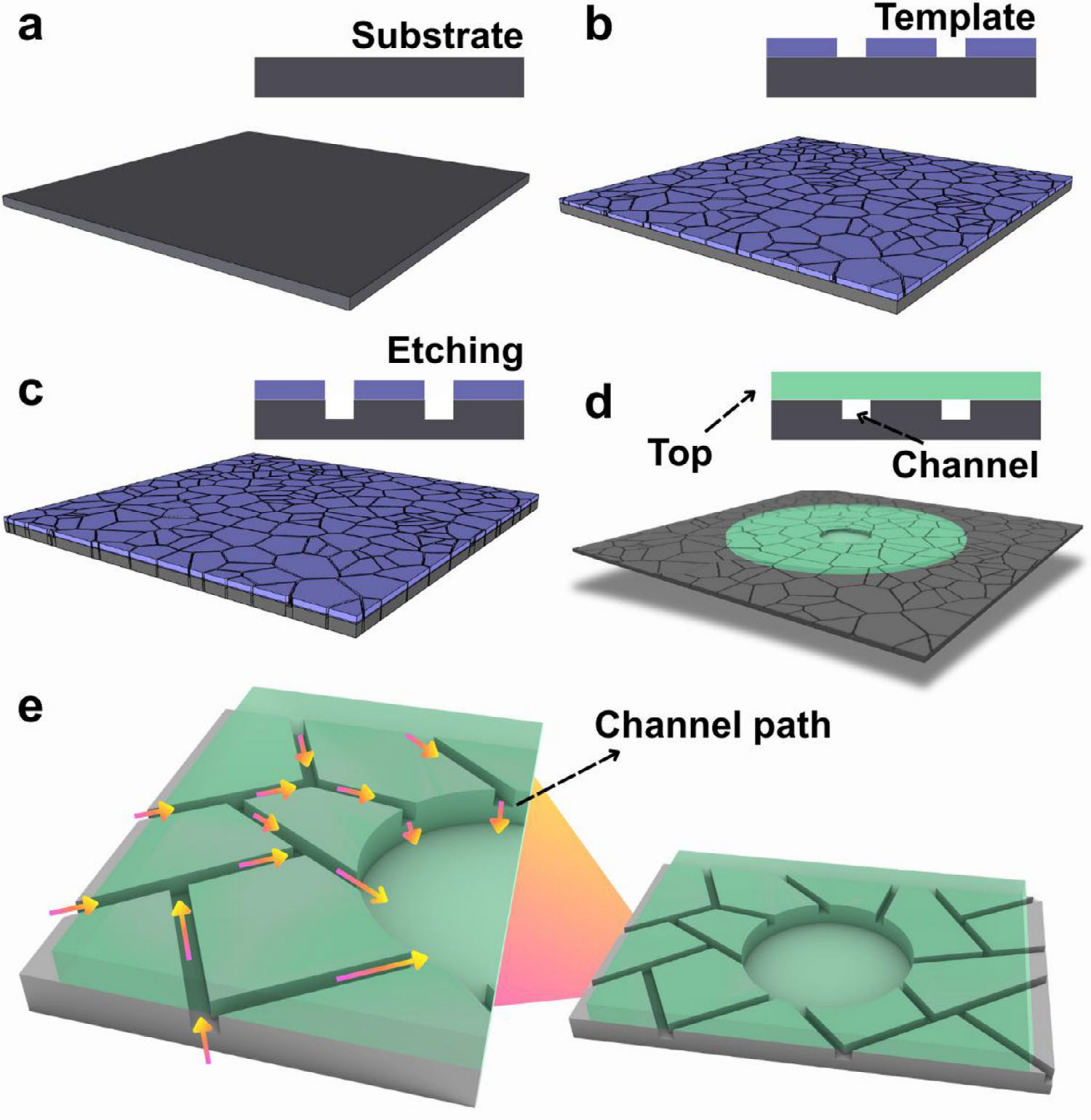
Schematic representing the process of nanofluidic device fabrication. (a) A clean 1 mm‐thick PETG substrate was prepared by 1 min oxygen‐plasma cleaning. (b) A precursor solution is spin‐coated over plasma‐treated substrate to obtain the crack template. (c) Channels etched inside the substrate by oxygen plasma using the template as a mask. (d) Top layer is thermally bonded over the channels to fabricate the nanochannel devices. (e) Enlarged schematic of the device near the exit hole and highlighted nanochannel pathways illustrating the routes available for fluid transport.

Our fabrication method for a nanofluidic device using crackle‐lithography is illustrated in Figure 1. The crackle precursors, such as diluted solutions of egg‐white and commercial SiO_2_ nanoparticle dispersions (~12 nm particle size, see materials and methods for further details), were spin‐coated over an oxygen‐plasma‐treated substrate (Figure 1a) to obtain an interconnected crack pattern (crackles) which are essentially down to the substrate (Figure 1b). The crackles emerge due to interfacial stress between particle‐particle and particle‐substrate during solvent evaporation [35]. By controlling the thickness of the crackle precursor, the crackle widths were precisely tuned (Figure S1). Besides being scalable, the crackle template approach offers various crackle dimensions with two different precursors, namely egg white and SiO_2_ nanoparticles, which are commercially available and inexpensive. The formed crackle template serves as a mask for the selective etching of the polymer substrate to create nano‐trenches (Figure 1c). The fabrication process was systematically optimized by exploring different precursors and substrates to achieve controlled crack formation suitable for nanofluidic channel development. Initially, egg white was employed as a natural crackle precursor, which produced interconnected crack networks a few micrometers wide (Figure S1). These microcracks served as an effective template for gold deposition, enabling the formation of conductive electrodes using the crack pattern as a mask (Figure S2). Interestingly, drying the template under vacuum conditions resulted in the appearance of radial crack patterns, indicating the strong influence of the drying environment on crack morphology (Figure S3).

To further reduce the feature dimensions into the nanometer regime, a SiO_2_ nanoparticle (SiO_2_‐NP) precursor was subsequently adopted as the crack‐forming template. Through systematic variation of spin parameters and dilution ratios, a spin speed of 1000 rpm and a dilution ratio of 2:3 (SiO_2_:DI water) were identified as optimal for uniform film formation and controllable crack widths (Figure S1).

In parallel, different polymer substrates were examined for their suitability in pattern transfer. Polyethylene terephthalate (PET) substrates were initially used owing to their compatibility with the oxygen plasma etching process using template as a mask, which enabled the formation of channels with tunable depth (Figure S4). However, polyethylene terephthalate glycol (PETG) was chosen as a suitable substrate for this device fabrication due to its ability to be etched with oxygen plasma, and its low bonding temperature (glass transition temperature) of 85 °C. To fabricate nanofluidic devices, the selectively etched PETG substrates pre‐drilled with a micro‐hole were thermally bonded with plain PETG substrates as depicted in scheme Figure 1d.

**FIGURE 2:**
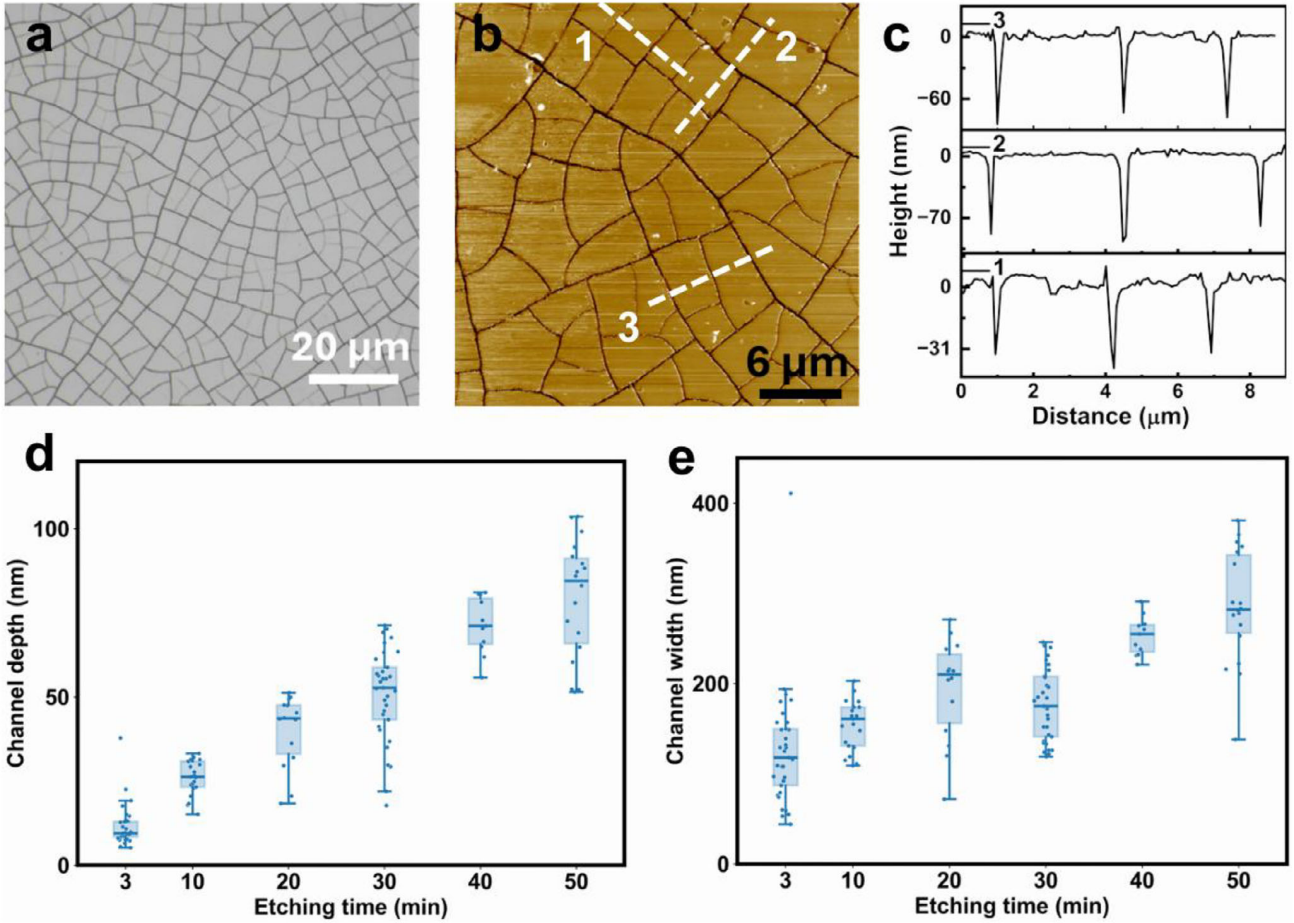
Depth and width tunability of nanochannels. (a) Optical microscopy image. (b) Atomic force microscopy image. (c) Representative height profile of nanochannels obtained after 30 min of etching. (d,e) Channel depth and width of PETG nanochannels as a function of oxygen‐plasma etching time.

The optical and atomic force microscopic images of PETG nanochannels etched for 30 min are depicted in Figure 2a,b. The depth and width of the nanofluidic channels could be varied with the duration of the oxygen plasma etching (Figure S5). For instance, 10 min etching produced ~30 nm‐deep channels, whereas for 50 min etching produced ~ 80 nm‐deep channels. To evaluate the reproducibility of the crackle‐derived nanochannels, we analyzed optical micrographs from multiple independently fabricated samples using connected component analysis. The largest connected component accounted for 99.5 ± 0.2% of the total network area, showing that the networks are highly interconnected and reproducible across samples (Figure S8). While the nanochannel width could be tuned by varying the etching time (Figure 2e), the overall crack‐area coverage only changes modestly from 17.1% to 20.9% across the investigated conditions. Similarly, the apparent tortuosity, estimated as the ratio between the transport‐path length and the corresponding straight‐line distance, remained fairly consistent, with an average value of 1.3 ± 0.2 (Figure S9). In addition, dead‐end pathways were quantified from the skeletonized crack networks by identifying terminal branches extending from each endpoint to the nearest junction. The dead‐end pathway fraction was calculated as the cumulative length of these terminal branches divided by the total skeletonized network length and was found to be only 0.3 ± 0.2% (Figure S10). This negligible dead‐end fraction is consistent with the connected‐component analysis and further confirms that the crackle‐derived nanochannel networks are highly interconnected.

Following the fabrication and bonding of the nanofluidic devices, their structural integrity and channel continuity were first confirmed through helium leak detection tests. The observed helium flow verified that the nanochannels were continuous, demonstrating the reliability of the crack‐lithography approach in producing functional conduits (Figure S11). AFM analysis performed after careful delamination further confirmed that the thermal bonding process did not measurably alter the nanochannel morphology or dimensions (Figure S12). With leak‐tight and structurally sound devices established, we turned our attention to their ability to support chemical reactions within these confined spaces. Using the same nanofluidic platform, we performed electroless metal deposition alongside optical measurements.

**FIGURE 3:**
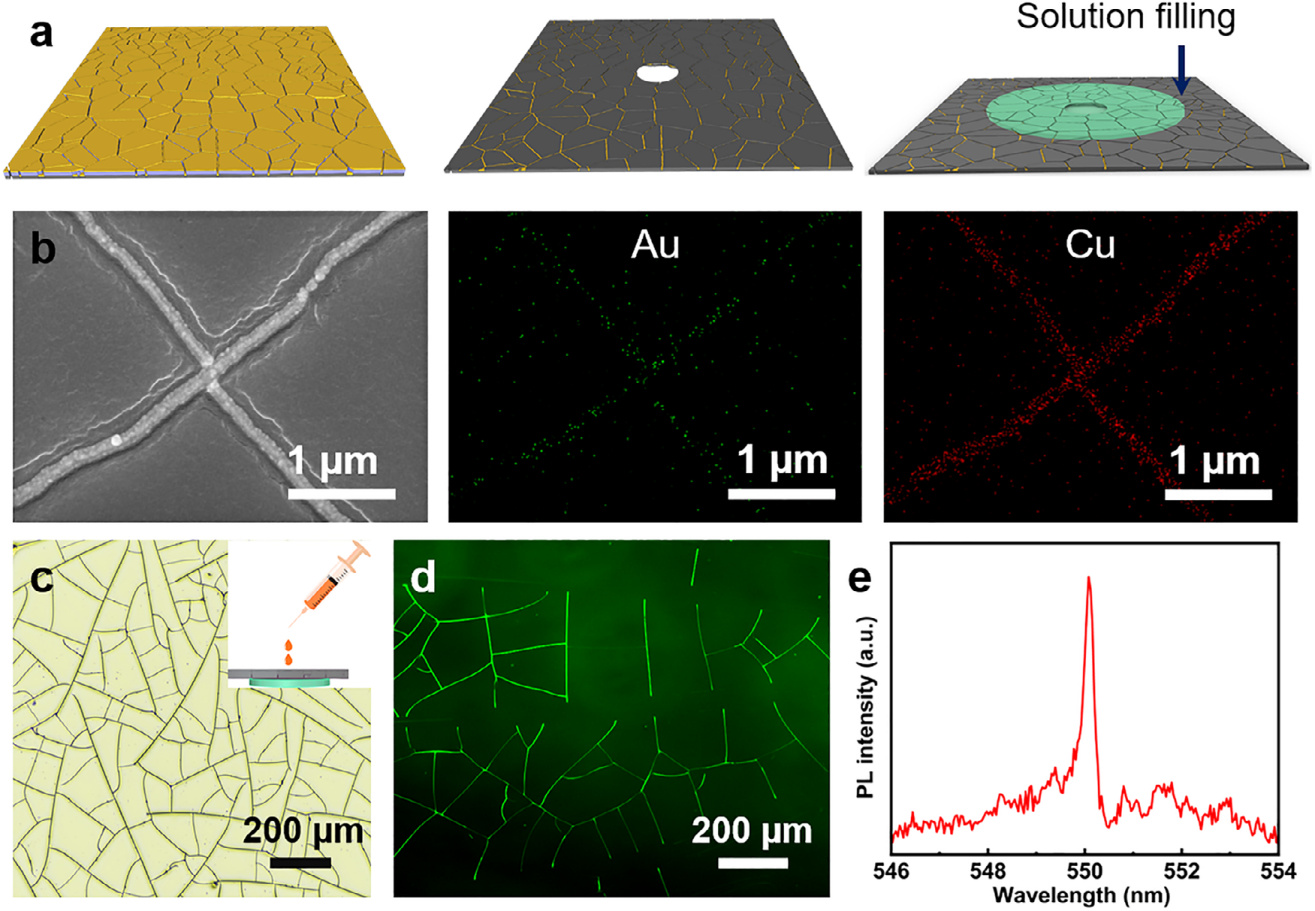
Copper electroless deposition and fluorescence microscopy. (a) Schematic representing the fabrication of nanochannel devices with an Au seed layer. (b) SEM image of the nanochannels after electroless deposition of copper with the corresponding EDS mapping of Au(Mα) and Cu(Lα) of the marked region. (c) Optical microscope image of the channels obtained using egg white as crackle precursor. Inset schematic showing the filling of Rhodamine 6G dye into the channels. (d) Fluorescence microscopic image showing the filling of the dye molecules in the interconnected channels. (e) PL spectroscopy confirming the presence of Rhodamine 6G in the channels.

Chemical reactions under nanoconfinement and catalytic confinements have been an exciting field of research due to their extensive applications from biological synthesis to chemical engineering [36, 37]. We demonstrate the catalytic activity in our nanochannels by electroless deposition of copper along the seed layer of gold inside the nanochannels. The gold seed layer was formed by thermal deposition of gold inside the nanochannels formed by plasma etching PETG substrates using SiO_2_ nanoparticle template as a mask and proceeding with the regular fabrication process to obtain the nanofluidic devices (Figure 3a). The Au seed layer provides essential nucleation sites for electroless Cu deposition, enabling localized Cu growth within the nanochannel network, while control experiments without Au showed no detectable Cu deposition along the channel pathways (Figure S14). The electroless plating solution (Cu precursor: formaldehyde, 10:1) was filled in the Au‐seeded nanochannels for a few minutes to obtain the deposition of Cu in the nanochannels. After optimizing the electroless Cu deposition process using open‐channel samples and SEM/EDS mapping (Figures S13 and S15), EDS analysis of the closed nanochannel devices confirmed Cu deposition along the Au seed layer inside the nanochannels, as shown in Figure 3b. The chemical state of the deposited Cu was further examined by XPS (Figure S16). The Cu 2p spectra showed only weak satellite features, while Cu LMM Auger analysis gave a modified Auger parameter of 1849.1 ± 0.2 eV, consistent with predominantly metallic Cu covered by a thin native Cu_2_O passivation layer rather than CuO/Cu(OH)_2_‐rich surface species [38, 39].

For better visualization of the channels under illumination, devices were fabricated using egg white as the crackle template precursor (Figure 3c) and filled with Rhodamine 6G (R6G) dye solution prepared in isopropyl alcohol (IPA). The channels were filled with R6G solution carefully using a syringe drop by drop (**inset** Figure 3c), and the presence of R6G in the channels was clearly evident from the fluorescent microscopic image under green light shown in Figure 3d and UV light in Figure S17. To further confirm the presence of R6G in the channels, photoluminescence spectroscopy was performed. An intense peak around 550 nm corresponding to the emission peak of R6G was obtained (Figure 3e). The transparent nature of the nanofluidic device as demonstrated, has huge potential in applications where molecules could be analyzed in situ during the reactions and even like lab on chip [40, 41]. After establishing the catalytic functionality and optical accessibility of the crackle‐derived nanochannels, we examined their ionic transport behavior to assess nanoscale electrochemical performance.

**FIGURE 4:**
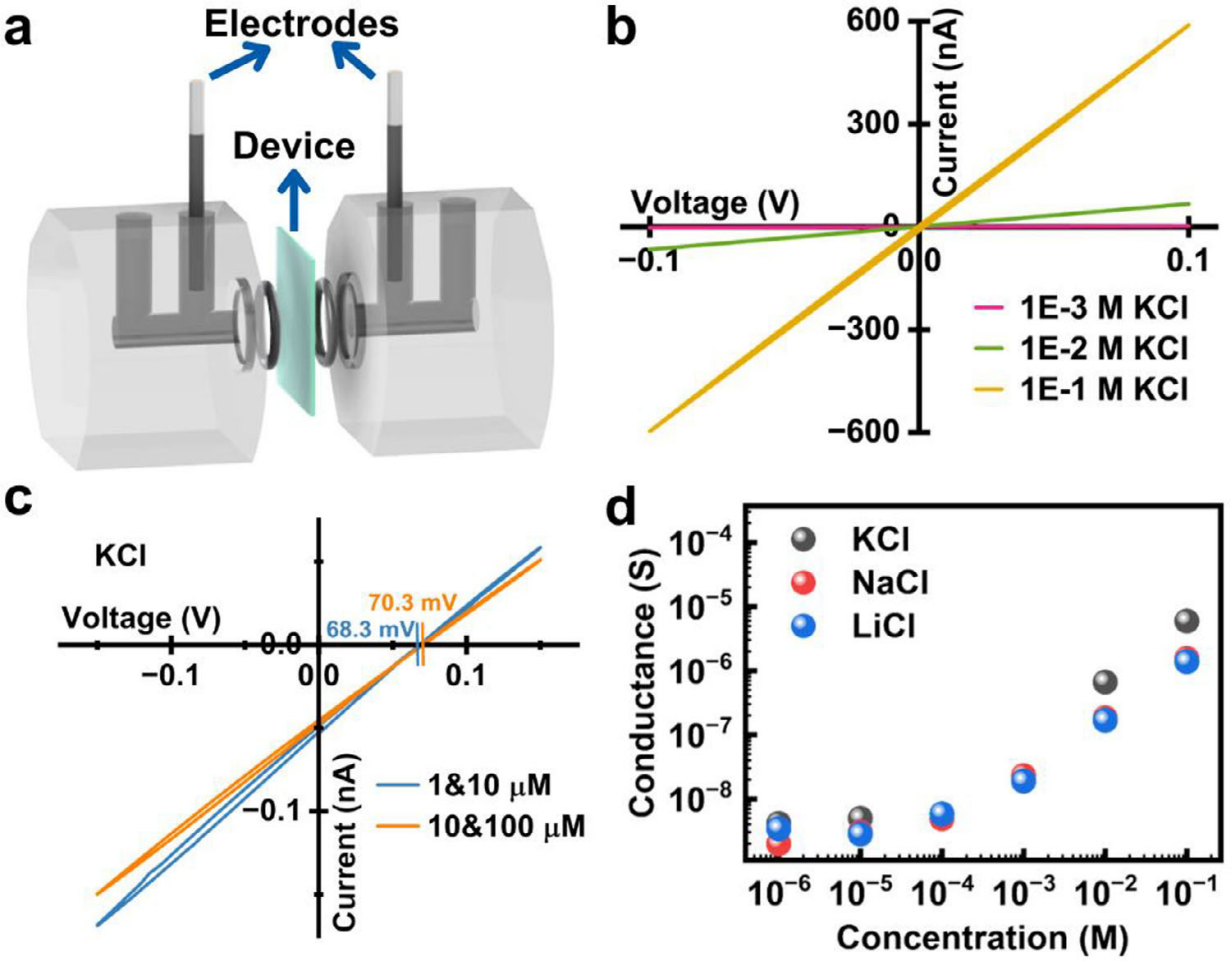
Ionic conductance measurements. (a) Schematic of the custom‐built electrochemical measurement setup showing the placement of electrodes and the nanofluidic device. (b) Current‐voltage (*I‐V*) response for KCl over a concentration range from 10^−3^ to 10^−1^ M. (c) I–V characteristics of drift‐diffusion experiments recorded using asymmetric KCl concentrations with a tenfold concentration gradient in the feed and permeate reservoirs (10 µM/1 µM and 100 µM/10 µM). (d) Conductance‐concentration characteristics of the PETG nanochannel device measured with monovalent electrolytes (KCl, NaCl, and LiCl).

The electrochemical measurements were performed using a custom‐built cell with symmetrical reservoirs and Ag/AgCl electrodes (Figure 4a). The current–voltage (*I–V*) characteristics were first recorded in monovalent electrolytes including KCl, NaCl, and LiCl over concentrations from 10^−6^ M to 10^−1^ M. At low applied voltages, the response in KCl exhibits Ohmic behavior (Figure 4b), consistent with nanofluidic ion transport [42, 43, 44]. The extracted conductance values were plotted as a function of electrolyte concentration in Figure 4d. Below 10^−4 ^M concentration, the conductance deviates from linear scaling due to surface‐charge‐dominated transport, where overlapping electric double layers enhance surface‐charge‐governed ion conduction [43, 45, 46, 47, 48]. This behavior is characteristic of confined nanofluidic systems and reflects the strong influence of surface chemistry under low ionic strength conditions, as depicted in the drift diffusion experiments in Figure 4c [49, 50]. After accounting for the Ag/AgCl redox potential, the effective membrane potential (*E_m_*) was 13–15 mV at low concentrations. Analysis using the Goldman‐Hodgkin‐Katz formalism yields a cation‐to‐anion mobility ratio *u*_+_/*u*_−_ of approximately 1.9 at 1–10 µM and ~2.1 at 10–100 µM KCl, confirming enhanced cation selectivity under surface‐charge governed transport [51, 52, 53]. In contrast, under bulk transport conditions at higher concentrations, E_m_ decreases to ~5 mV, corresponding to a mobility ratio approaching unity, consistent with diffusion‐dominated ionic conduction (Figure S18) [43, 44].

Building on the established ion transport characteristics, we extended the applied potential window to explore whether the PETG nanochannels exhibit memristive signatures relevant to neuromorphic computing. Such systems are analogous to biological synapses, where the conductance state encodes past electrical stimuli and governs the response to new inputs [54]. In nanofluidic memristors, this behavior arises from the slow evolution of internal ionic configurations, including ion accumulation, depletion, adsorption, and surface charge modulation, which causes the current at a given voltage to depend on the prior voltage and ionic history. This history‐dependent transport manifests as pinched hysteresis loops in *I–V* responses [55].

**FIGURE 5:**
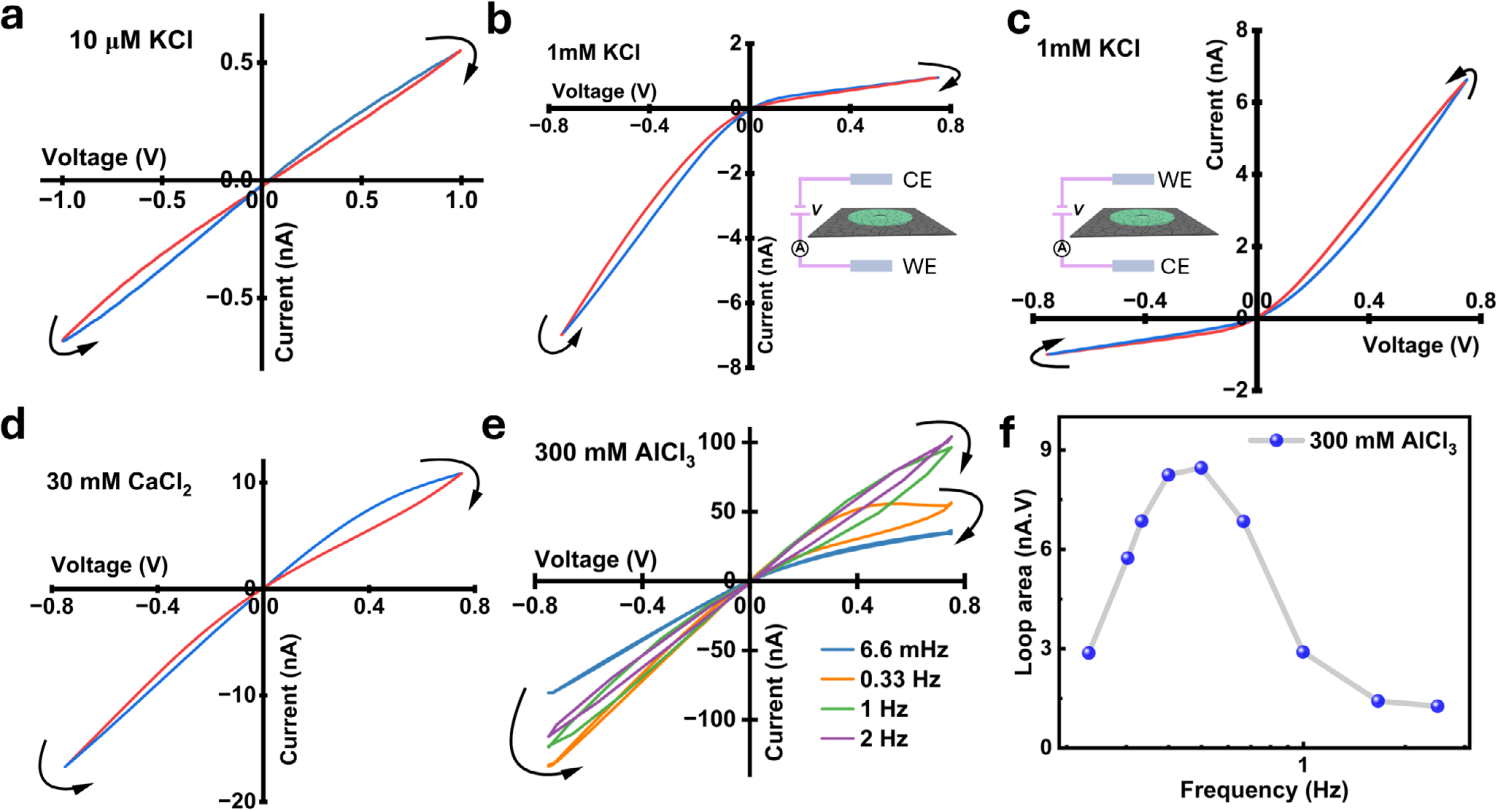
Ionic memory measurements of PETG nanochannel devices. (a) M1‐type memristive *I–V* loop obtained in 10 µM KCl. (b,c) Effect of electrode orientation on the *I‐V* response in 1 mM KCl. When the working electrode (WE) is placed on the hole side and the counter electrode (CE) on the nanochannel side, the device shows an M1‐type loop (b), whereas reversing the electrode configuration produces an M3‐type loop (c). The insets show the corresponding electrode configurations. (d) M1‐type clockwise memristive loop obtained in 30 mM CaCl_2_. (e) Frequency‐dependent *I–V* characteristics recorded in 300 mM AlCl_3_ at different voltage sweep rates. (f) *I–V* loop area as a function of applied voltage frequency for 300 mM AlCl_3_ salt solution.

By systematically varying electrolyte type and concentration, two different types of memristive loops were obtained (Figure 5a–d). For 10 µM KCl, the device exhibits an M1‐type memristive loop (Figure 5a). At this low ionic strength, surface‐charge effects dominate the transport, consistent with the cation‐selective behavior observed in the drift‐diffusion measurements. The delayed redistribution of ions under cyclic bias produces ion accumulation and depletion zones that evolve with the applied voltage history, giving rise to the observed M1‐type hysteresis. As the KCl concentration is increased, electrostatic screening becomes stronger, the transport becomes more bulk‐like, and the hysteresis is progressively reduced.

The memory response can also be tuned by changing the electrode configuration. In 1 mM KCl, placing the working electrode on the hole side and the counter electrode on the nanochannel side produces an M1‐type loop (Figure 5b). Reversing the electrode configuration changes the direction of ionic drift across the asymmetric nanochannel geometry and converts the response to an M3‐type loop (Figure 5c). This inversion is consistent with a reversal of the ion accumulation and depletion profiles during the voltage cycle. Similar polarity‐dependent switching between memristive loop types has recently been observed in asymmetric charged nanofluidic channel systems, where electrode reversal modifies the direction of ionic asymmetry without changing the electrolyte, surface chemistry, or device structure [56].

For 30 mM CaCl_2_, an M1‐type loop is also observed (Figure 5d). Although Ca^2^^+^ can interact more strongly with negatively charged surface sites than monovalent K^+^, the surface‐charge contribution remains sufficiently important to support delayed ion accumulation and depletion. This suggests that divalent cations modify the local charge environment but do not completely suppress the ionic relaxation processes responsible for the memory response.

In 300 mM AlCl_3_, the device continues to exhibit a memristive response despite the high ionic strength and strong ion–surface interactions (Figure 5e). In this case, the hysteresis is unlikely to arise from the same dilute‐limit surface‐charge mechanism observed in 10 µM KCl. Instead, the response is likely governed by a charge‐regulated transport state involving strong Al^3^^+^–surface interactions, enhanced electrostatic screening, and partial compensation of the native negative surface charge. The frequency‐dependent *I–V* characteristics and corresponding loop‐area variation (Figures 5e,f and S20) further indicate that the memory response is controlled by finite ionic relaxation times. At frequencies where the voltage sweep is comparable to the characteristic ion redistribution timescale, the hysteresis is enhanced, whereas very slow or very fast sweeps reduce the loop area because the ionic configuration either relaxes nearly completely or cannot follow the applied voltage efficiently. These results show that crackle‐derived PETG nanochannels support intrinsic ionic memory behaviour that can be tuned by electrolyte composition, ionic valency, concentration, applied frequency, and electrode configuration. The ability to obtain and invert memristive loop types in a low‐cost, transparent, and scalable plastic nanofluidic platform highlights the potential of these devices for iontronic memory and neuromorphic applications.

To confirm that the observed memristive signatures are intrinsic to the nanochannels and do not arise from electrode‐electrolyte interfacial processes [57], control experiments were performed by replacing Ag/AgCl electrodes with inert platinum (Pt) electrodes. The resulting *I–V* characteristics remained comparable and confirm that the memristive behavior is independent of the electrode material (Figure S21). These results establish that the polymer‐based crackle‐derived nanochannels support intrinsic ion‐driven resistive switching governed by internal ionic dynamics. The ability to engineer a low‐cost, transparent, and scalable plastic platform highlights the potential of these systems as building blocks for ionic circuits.

## 3 Conclusions

In this work, we have successfully demonstrated crackle‐lithography as a simple, low‐cost, and scalable approach for fabricating plastic nanofluidic devices. By exploiting naturally occurring crack patterns as etching masks, we successfully produced nanochannels with tuneable dimensions on PETG substrates and subsequently assembled them into functional nanofluidic devices. The devices were shown to support confined catalytic reactions, as demonstrated by copper electroless deposition, and the optical interrogation capabilities of these devices were evidenced through fluorescence microscopy of molecules inside the channels. Furthermore, electrochemical measurements revealed intrinsic memristive behavior with distinct loop types, highlighting their potential for neuromorphic and information processing applications.

Together, these findings establish crackle‐lithography as a powerful alternative to conventional nanofabrication techniques. Its compatibility with inexpensive precursors, high throughput, and capacity for device upscaling make it particularly useful for industrial translation. Beyond nanofluidics, this strategy fosters possibilities for developing multifunctional platforms in areas such as lab‐on‐chip systems, biosensing, and molecular electronics. Future efforts will focus on further refining crack pattern control, extending substrate versatility, and integrating device architectures to fully harness the potential of this unconventional yet robust fabrication approach.

## 4 Experimental

### 4.1 Materials

PETG substrates were cut from clear plastic sheets (RS PRO, product no. 704–8175, 1250 mm × 1250 mm × 1 mm). PET substrates were purchased from Lloyd Paton (Product No. QR0031) as A4‐transparent sheets. A colloidal silica nanoparticle dispersion (LUDOX HS‐40) was obtained from Sigma‐Aldrich. Hydrophilic polytetrafluoroethylene (PTFE) syringe filters (0.22 µm pore size, ALLPURE, China) were used to filter the crackle precursors. Isopropyl alcohol (≥99.8%), potassium chloride, lithium chloride, sodium chloride, aluminum chloride, and sodium hydroxide were purchased from Fisher Scientific. Sodium potassium tartrate, formaldehyde (36.5%–38%), and rhodamine 6G were obtained from Sigma‐Aldrich. Copper(II) sulphate was purchased from Acros Organics, and hydrogen chloride from Thermo Scientific Acros. All chemicals and materials were used as received, without further purification.

### 4.2 Characterization Methods

Optical microscopy was performed using a Nikon Eclipse LV100ND microscope. Atomic force microscopy (AFM) was performed on Dimension Icon and Dimension FastScan systems (Bruker, USA) operated in tapping and contact modes. Scanning electron microscopy (SEM) of Cu‐deposited channels was carried out on a Zeiss ULTRA 55 (2600 Series). Prior to imaging, the top layer was peeled off, and a 9 nm Pt film was deposited by electron‐beam evaporation to ensure conductivity; images were acquired with an InLens detector at an EHT of 5.00 kV. Energy‐dispersive x‐ray spectroscopy (EDX) was conducted using an Oxford X‐Max 80 detector (Oxford Instruments, UK). Fluorescence microscopy was performed on a Nikon TE2000‐U (Nikon, Japan) under UV and green illumination using a 10× objective. Photoluminescence (PL) spectra were obtained with inVia confocal microscope (Renishaw, UK) using 532 nm excitation and a 50× objective. X‐ray Photoelectron Spectroscopy (XPS) was performed using an Axis Supra+ spectrometer (Kratos Analytical, Manchester, United Kingdom) using monochromated Al Kα radiation (1486.6 eV, 20 mA emission at 300 W, spot size 1 mm) with a base vacuum pressure of 5×10^−9^ mbar. Charge neutralization was achieved using a low‐energy electron flood source.

### 4.3 Preparation of SiO_2_ Crackle Precursor

SiO_2_ crackle precursor was obtained by diluting the commercial SiO_2_ nanoparticle dispersion with DI water (SiO_2_:H_2_O in 1:1.5). The precursor, filtered twice using 220 nm hydrophilic PTFE (HPPTFE) syringe filter, was spin‐coated over the plasma‐treated PETG substrates with a 1000 rpm spin rate.

### 4.4 Preparation of Egg‐White Crackle Precursor

For the egg white (EW) precursor, fresh hen eggs were cracked, and the fluid part was carefully sieved out and left undisturbed for a few hours. The supernatant layer was diluted with DI water (EW:H_2_O in 2:1) and drop‐coated over plasma‐treated substrates.

### 4.5 Bonding

Owing to the low (glass transition temperature) T_G_ of PETG, the same material can be used as the top cover as well. An 8 mm circular disc of PETG was used as the top cover, and a 0.6 mm hole was drilled in the etched substrate with the utmost caution to avoid deformation of the substrate, as it reduces the area of contact between the substrate and the top cover. The oxygen plasma treatment was done for the surfaces of both the substrate and the top cover to improve adhesion. In the next step, the substrate and top cover were bonded under pressure by stacking the device between two glass cover slips and placing it over a pre‐heated hotplate at 85°C under a load of approximately 1 kg. It is found that 30 min is the optimum time required for complete bonding.

### 4.6 Copper Electroless Deposition

The copper electroless plating solution is a composition of two solutions mixed in a ratio of 10:1. The solution A is composed of 300 mg of CuSO_4_, 1.4 g of sodium potassium tartrate, and 400 mg of NaOH mixed in 10 mL deionized water whereas the solution B consists of formaldehyde solution (37.2 wt.%). The solution was poured into the nanochannels through the sides of the device for a few minutes and washed away with water.

### 4.7 Electrochemical Experiments

Electrochemical measurements were carried out in a custom polyether ether ketone (PEEK) cell comprising two reservoirs (≈2.5 mL each) and a dual O‐ring seal to position and secure the device (Figure 4a). Each reservoir incorporated ports for electrode insertion. Prior to each experiment, the cell body and O‐rings were rinsed with deionized (DI) water and dried under a nitrogen stream. Devices were then mounted, and the reservoirs were filled with electrolytes prepared in Milli‐Q DI water (resistivity ≈18 MΩ cm), dispensed gently with a 3 mL pipette to minimize bubble formation. Current–voltage (I–V) characteristics were recorded using a Keithley 2636B SourceMeter and a BioLogic SP‐300 potentiostat/galvanostat with standard Ag/AgCl electrodes. Data were acquired with LabVIEW 2019 (Keithley 2636B) and EC‐Lab (BioLogic SP‐300).

## Author Contributions

B.R. and A.K conceived the idea and co‐directed the project with S.K. T.R.D.. S.K., B.R., and A.K., designed the experiments. T.R.D. and S.K. fabricated the devices and carried out the copper electroless deposition. T.R.D. performed all characterization experiments. M.S.M. and T.R.D. conducted the fluorescence microscopy measurements and XPS analysis. S.V.P. and T.R.D. performed all electrochemical experiments. H.W. and T.R.D. carried out the computational analysis of the channels. T.R.D., B.R., and A.K. wrote the manuscript with input from S.K. and S.V.P., and all authors contributed to the discussions.

## Conflicts of Interest

The authors declare no conflicts of interest

## Supporting information




**Supporting File**: advs76980‐sup‐0001‐SuppMat.pdf.

## Data Availability

The data that supports the findings of this study are available in the supplementary material of this article.

## References

[1] E. Secchi , S. Marbach , A. Niguès , D. Stein , A. Siria , and L. Bocquet , “Massive Radius‐Dependent Flow Slippage in Carbon Nanotubes,” Nature 537, no. 7619 (2016): 210–213, 10.1038/nature19315. 27604947 PMC5015706

[2] R. H. Tunuguntla , R. Y. Henley , Y.‐C. Yao , T. A. Pham , M. Wanunu , and A. Noy , “Enhanced Water Permeability and Tunable Ion Selectivity in Subnanometer Carbon Nanotube Porins,” Science 357, no. 6353 (2017): 792–796, 10.1126/science.aan2438. 28839070

[3] J. K. Holt , H. G. Park , Y. Wang , et al., “Fast Mass Transport Through Sub‐2‐Nanometer Carbon Nanotubes,” Science 312, no. 5776 (2006): 1034–1037, 10.1126/science.1126298. 16709781

[4] L. Fumagalli , A. Esfandiar , R. Fabregas , et al., “Anomalously Low Dielectric Constant of Confined Water,” Science 360, no. 6395 (2018): 1339–1342, 10.1126/science.aat4191. 29930134

[5] A. Keerthi , A. K. Geim , A. Janardanan , et al., “Ballistic Molecular Transport Through Two‐Dimensional Channels,” Nature 558, no. 7710 (2018): 420–424, 10.1038/s41586-018-0203-2. 29925968

[6] G. M. Preston , T. P. Carroll , W. B. Guggino , and P. Agre , “Appearance of Water Channels in Xenopus Oocytes Expressing Red Cell CHIP28 Protein,” Science 256, no. 5055 (1992): 385–387, 10.1126/science.256.5055.385. 1373524

[7] W. A. Catterall , “Ion Channel Voltage Sensors: Structure, Function, and Pathophysiology,” Neuron 67 (2010): 915–928, 10.1016/j.neuron.2010.08.021. 20869590 PMC2950829

[8] B. Choat , S. Jansen , M. A. Zwieniecki , E. Smets , and N. M. Holbrook , “Changes in Pit Membrane Porosity Due to Deflection and Stretching: The Role of Vestured Pits,” Journal of Experimental Botany 55, no. 402 (2004): 1569–1575, 10.1093/jxb/erh173. 15181107

[9] G. B. West , J. H. Brown , and B. J. Enquist , “The Fourth Dimension of Life: Fractal Geometry and Allometric Scaling of Organisms,” Science 284, no. 5420 (1999): 1677–1679, 10.1126/science.284.5420.1677. 10356399

[10] B. Blonder , C. Violle , L. P. Bentley , and B. J. Enquist , “Venation Networks and the Origin of the Leaf Economics Spectrum,” Ecology Letters 14, no. 2 (2011): 91–100, 10.1111/j.1461-0248.2010.01554.x. 21073643

[11] N. Bowden , S. Brittain , A. G. Evans , J. W. Hutchinson , and G. M. Whitesides , “Spontaneous Formation of Ordered Structures in Thin Films of Metals Supported on an Elastomeric Polymer,” Nature 393, no. 6681 (1998): 146–149, 10.1038/30193.

[12] G. M. Whitesides , J. P. Mathias , and C. T. Seto , “Molecular Self‐Assembly and Nanochemistry: A Chemical Strategy for the Synthesis of Nanostructures,” Science 254, no. 5036 (1991): 1312–1319, 10.1126/science.1962191. 1962191

[13] B. Radha and G. U. Kulkarni , “A Modified Micromolding Method for Sub‐100‐nm Direct Patterning of Pd Nanowires,” Small 5, no. 20 (2009): 2271–2275, 10.1002/smll.200900768. 19582731

[14] H. Kim , M. K. Abdelrahman , J. Choi , et al., “From Chaos to Control: Programmable Crack Patterning With Molecular Order in Polymer Substrates,” Advanced Materials 33, no. 22 (2021): 2008434, 10.1002/adma.202008434. 33860580

[15] L. Goehring , R. Conroy , A. Akhter , W. J. Clegg , and A. F. Routh , “Evolution of Mud‐Crack Patterns During Repeated Drying Cycles,” Soft Matter 6, no. 15 (2010): 3562–3567, 10.1039/b922206e.

[16] K. Pasricha , U. Wad , R. Pasricha , and S. Ogale , “Parametric Dependence Studies on Cracking of Clay,” Physica A: Statistical Mechanics and its Applications 388, no. 8 (2009): 1352–1358, 10.1016/j.physa.2008.12.039.

[17] A. F. Martins , N. C. Bennett , S. Clavel , et al., “Locally‐Curved Geometry Generates Bending Cracks in the African Elephant Skin,” Nature Communications 9, no. 1 (2018): 1–8, 10.1038/s41467-018-06257-3. PMC6168576 30279508

[18] M. C. Milinkovitch , L. Manukyan , A. Debry , et al., “Crocodile Head Scales Are Not Developmental Units But Emerge From Physical Cracking,” Science 339, no. 6115 (2013): 78–81, 10.1126/science.1226265. 23196908

[19] J. Jung , K. K. Kim , Y. D. Suh , S. Hong , J. Yeo , and S. H. Ko , “Recent Progress in Controlled Nano/Micro Cracking as an Alternative Nano‐Patterning Method for Functional Applications,” Nanoscale Horizons 5, no. 7 (2020): 1036–1049, 10.1039/D0NH00241K. 32469038

[20] M. Kim , D.‐J. Kim , D. Ha , and T. Kim , “Cracking‐Assisted Fabrication of Nanoscale Patterns for Micro/Nanotechnological Applications,” Nanoscale 8, no. 18 (2016): 9461–9479, 10.1039/C5NR06266G. 26691345

[21] S. Kiruthika , K. D. M. Rao , A. Kumar , R. Gupta , and G. U. Kulkarni , “Metal Wire Network Based Transparent Conducting Electrodes Fabricated Using Interconnected Crackled Layer as Template,” Materials Research Express 1, no. 2 (2014): 026301, 10.1088/2053-1591/1/2/026301.

[22] J. H. Lee , B. Chung , S. Park , H. C. Moon , and D. H. Lee , “Fabrication of Grid‐Type Transparent Conducting Electrodes Based on Controlled Mechanical Fracture,” Macromolecular Research 26, no. 2 (2018): 157–163, 10.1007/s13233-018-6026-z.

[23] O. Dalstein , E. Gkaniatsou , C. Sicard , et al., “Evaporation‐Directed Crack‐Patterning of Metal–Organic Framework Colloidal Films and Their Application as Photonic Sensors,” Angewandte Chemie International Edition 56, no. 45 (2017): 14011–14015, 10.1002/anie.201706745. 28940925

[24] S. Kiruthika , S. Singh , and G. U. Kulkarni , “Large area transparent ZnO Photodetectors With Au wire network electrodes,” RSC Advances 6 (2016): 44668–44672, 10.1039/c6ra07118j.

[25] S. Kiruthika and G. U. Kulkarni , “Smart Electrochromic Supercapacitors Made of Metal Mesh Electrodes With Polyaniline as Charge Storage Indicator,” Energy Technology 8, no. 5 (2020): 1901364, 10.1002/ente.201901364.

[26] S. Kiruthika , C. Sow , and G. Kulkarni , “Transparent and Flexible Supercapacitors With Networked Electrodes,” Small 13, no. 40 (2017): 1701906, 10.1002/smll.201701906. 28834115

[27] I. Mondal , S. Kiruthika , M. K. Ganesha , et al., “ITO‐Free Large Area PDLC Smart Windows: A Cost‐Effective Fabrication Using Spray Coated SnO_2_ on an Invisible Al Mesh,” Journal of Materials Chemistry A 9, no. 40 (2021): 23157–23168, 10.1039/D1TA05820G.

[28] S. Gong , L. W. Yap , B. Zhu , et al., “Local Crack‐Programmed Gold Nanowire Electronic Skin Tattoos for In‐Plane Multisensor Integration,” Advanced Materials 31, no. 41 (2019): 1903789, 10.1002/adma.201903789. 31448484

[29] D. Kang , P. V. Pikhitsa , Y. W. Choi , et al., “Ultrasensitive Mechanical Crack‐Based Sensor Inspired by the Spider Sensory System,” Nature 516, no. 7530 (2014): 222–226, 10.1038/nature14002. 25503234

[30] D.‐S. Kim , Y. W. Choi , A. Shanmugasundaram , et al., “Highly Durable Crack Sensor Integrated With Silicone Rubber Cantilever for Measuring Cardiac Contractility,” Nature Communications 11, no. 1 (2020): 535, 10.1038/s41467-019-14019-y. PMC6985253 31988308

[31] K. H. Nam , I. H. Park , and S. H. Ko , “Patterning by Controlled Cracking,” Nature 485, no. 7397 (2012): 221–224, 10.1038/nature11002. 22575963

[32] B.‐Y. Xu , J.‐J. Xu , X.‐H. Xia , and H.‐Y. Chen , “Large Scale Lithography‐Free Nano Channel Array on Polystyrene,” Lab on a Chip 10, no. 21 (2010): 2894–2901, 10.1039/c005245k. 20922216

[33] M. Kim and T. Kim , “Crack‐Photolithography for Membrane‐Free Diffusion‐Based Micro/Nanofluidic Devices,” Analytical Chemistry 87, no. 22 (2015): 11215–11223, 10.1021/acs.analchem.5b02028. 26140611

[34] M. Kim , D. Ha , and T. Kim , “Cracking‐Assisted Photolithography for Mixed‐Scale Patterning and Nanofluidic Applications,” Nature Communications 6, no. 1 (2015): 6247, 10.1038/ncomms7247. 25692794

[35] W. P. Lee and A. F. Routh , “Why Do Drying Films Crack?,” Langmuir 20, no. 23 (2004): 9885–9888, 10.1021/la049020v. 15518466

[36] Y. Shen , X. Wang , J. Lei , S. Wang , Y. Hou , and X. Hou , “Catalytic Confinement Effects in Nanochannels: From Biological Synthesis to Chemical Engineering,” Nanoscale Advances 4, no. 6 (2022): 1517–1526, 10.1039/D2NA00021K. 36134369 PMC9418946

[37] A. B. Grommet , M. Feller , and R. Klajn , “Chemical Reactivity Under Nanoconfinement,” Nature Nanotechnology 15, no. 4 (2020): 256–271, 10.1038/s41565-020-0652-2. 32303705

[38] M. C. Biesinger , “Advanced Analysis of Copper X‐Ray Photoelectron Spectra,” Surface and Interface Analysis 49, no. 13 (2017): 1325–1334, 10.1002/sia.6239.

[39] P. Bébin and R. E. Prud'homme , “Comparative XPS Study of Copper, Nickel, and Aluminum Coatings on Polymer Surfaces,” Chemistry of Materials 15, no. 4 (2003): 965–973, 10.1021/cm020599x.

[40] L. M. Bellan , T. Kniazeva , E. S. Kim , et al., “Fabrication of a Hybrid Microfluidic System Incorporating Both Lithographically Patterned Microchannels and a 3D Fiber‐Formed Microfluidic Network,” Advanced Healthcare Materials 1, no. 2 (2012): 164–167, 10.1002/adhm.201100052. 22708076 PMC3374650

[41] K. S. Phillips , H. H. Lai , E. Johnson , C. E. Sims , and N. L. Allbritton , “Continuous Analysis of Dye‐Loaded, Single Cells on a Microfluidic Chip,” Lab on a Chip 11, no. 7 (2011): 1333–1341, 10.1039/c0lc00370k. 21327264 PMC3462073

[42] T. Emmerich , K. S. Vasu , A. Niguès , et al., “Enhanced Nanofluidic Transport in Activated Carbon Nanoconduits,” Nature Materials 21, no. 6 (2022): 696–702, 10.1038/s41563-022-01229-x. 35422506

[43] A. Esfandiar , B. Radha , F. C. Wang , et al., “Size Effect in Ion Transport Through Angstrom‐Scale Slits,” Science 358, no. 6362 (2017): 511–513, 10.1126/science.aan5275. 29074772

[44] A. Bhardwaj , R. K. Gogoi , W. J. Howard , et al., “Ultramicrotomy‐Assisted Fabrication of Nanochannels for Efficient Ion Transport and Energy Generation,” Advanced Functional Materials 34, no. 39 (2024): 2401988, 10.1002/adfm.202401988.

[45] L. Bocquet and E. Charlaix , “Nanofluidics, From Bulk to Interfaces,” Chemical Society Review 39, no. 3 (2010): 1073–1095, 10.1039/B909366B. 20179826

[46] T. Mouterde , A. Keerthi , A. R. Poggioli , et al., “Molecular Streaming and Its Voltage Control in Ångström‐Scale Channels,” Nature 567, no. 7746 (2019): 87–90, 10.1038/s41586-019-0961-5. 30842639

[47] F. Baldessari , “Electrokinetics in Nanochannels,” Journal of Colloid and Interface Science 325, no. 2 (2008): 526–538, 10.1016/j.jcis.2008.06.007. 18639883

[48] Q. Pu , J. Yun , H. Temkin , and S. Liu , “Ion‐Enrichment and Ion‐Depletion Effect of Nanochannel Structures,” Nano Letters 4, no. 6 (2004): 1099–1103, 10.1021/nl0494811.

[49] J. A. Allegretto , G. Laucirica , A. L. Huamani , et al., “Manipulating Ion Transport Regimes in Nanomembranes via a 'Pore‐in‐Pore' Approach Enabled by the Synergy of Metal–Organic Frameworks and Solid‐State Nanochannels,” ACS Nano 18, no. 28 (2024): 18572–18583, 10.1021/acsnano.4c04435. 38941562

[50] G. Laucirica , J. A. Allegretto , M. F. Wagner , et al., “Switchable Ion Current Saturation Regimes Enabled via Heterostructured Nanofluidic Devices Based on Metal–Organic Frameworks,” Advanced Materials 34, no. 51 (2022): 2207339, 10.1002/adma.202207339. 36239253

[51] D. E. Goldman , “Potential, Impedance, and Rectification in Membranes,” Journal of General Physiology 27, no. 1 (1943): 37–60, 10.1085/jgp.27.1.37. 19873371 PMC2142582

[52] A. L. Hodgkin and B. Katz , “The Effect of Sodium Ions on the Electrical Activity of the Giant Axon of the Squid,” The Journal of Physiology 108, no. 1 (1949): 37–77, 10.1113/jphysiol.1949.sp004310. PMC1392331 18128147

[53] R. B. Schoch , J. Han , and P. Renaud , “Transport Phenomena in Nanofluidics,” Reviews of Modern Physics 80, no. 3 (2008): 839–883, 10.1103/RevModPhys.80.839.

[54] S. V. Pandey , K. V. Saurav , A. Ismail et al., “Nanofluidic Ionic Memory for Next‐Generation Computing,” Nature Reviews Materials (2026), 10.1038/s41578-026-00919-1.

[55] P. Robin , T. Emmerich , A. Ismail , et al., “Long‐Term Memory and Synapse‐Like Dynamics in Two‐Dimensional Nanofluidic Channels,” Science 379, no. 6628 (2023): 161–167, 10.1126/science.adc9931. 36634187

[56] S. Yadav , R. K. Gogoi , A. Lokhandwala , S. V. Pandey , A. Bhardwaj , S. D. Gupta , et al., “Two‐Dimensional Clay Channels for Tunable Nanofluidic Memristor,” Communications Materials (2026), 10.1038/s43246-026-01307-6.

[57] S. V. Pandey , K. V. Saurav , S. Goutham , A. Sharma , A. Keerthi , and B. Radha , “Ionic Memory or Electrode Artefacts? A Systematic Assessment of Nanofluidic Memristors,” Faraday Discussions (2026), 10.1039/d5fd00135h. 42093527

