# Supplementary material for: Scalable Crackle‐Lithography for Multifunctional Enclosed Plastic Nanofluidic Devices
Source: Adv Sci (Weinh). 2026 Aug 6:e76980. Online ahead of print. doi: 10.1002/advs.76980 (PMC13445325; doi:10.1002/advs.76980)
Supplement: Supplementary file 1 [file ADVS-9999-e76980-s001.pdf]

# Supporting Information

# Scalable Crackle-Lithography for Multifunctional Enclosed Plastic Nanofluidic Devices

Tirumala Rao Dumpala[1,2,3], S. Kiruthika[4], Siddhi Vinayak Pandey[1,3], Junyang Zhang[2], Solleti Goutham[1,3], Marcos Vinicius Surmani Martins[1,3], Harry Whittingham[1], Boya Radha[1,3,5*], Ashok Keerthi[2,3,5*]

[1]Department of Physics and Astronomy, School of Natural Sciences, The University of Manchester, Oxford Road, Manchester, M13 9PL, UK.
[2]Department of Chemistry, School of Natural Sciences, The University of Manchester, Oxford Road, Manchester, M13 9PL, UK.
[3]National Graphene Institute, The University of Manchester, Booth Street East, Manchester, M13 9PL, UK.
[4]Department of Physics, School of Electrical and Electronics Engineering, SASTRA Deemed to be University, Thanjavur, Tamil Nadu, India.
[5]Photon Science Institute, The University of Manchester, Oxford Road, Manchester, M13 9PL, UK.

*Correspondence: radha.boya@manchester.ac.uk and ashok.keerthi@manchester.ac.uk

**Supplementary information**

## Data and Code Availability

The data that supports the findings of this study are available in the supplementary material of this article. The simulation code used to computationally analyse the depth and width of the channels is available in a public GitHub repository [https://github.com/tirumalarao439/Plastic-nanofluidic-devices-fabricated-using-unconventional-crackle-lithography.git].

**List of supplementary figures**


- Figure S1. Control of crack patterns
- Figure S2. Gold conductive electrode fabricated using a crackle template mask
- Figure S3. Radially guided cracks
- Figure S4. Depth analysis of PET nanochannels
- Figure S5. AFM analysis of channel depth and width
- Figure S6. Sequential image processing for width analysis of nanochannels
- Figure S7. Width analysis of nanochannels *via* sequential image processing
- Figure S8. Image-processing workflow used for crack-area and connectivity analysis of the crackle-patterned network
- Figure S9. Sequential image-processing workflow for quantitative crack-network transport analysis
- Figure S10. Dead-end path analysis of crack-defined nanochannel networks
- Figure S11. Helium leak measurements
- Figure S12. Stability of nanochannels after top-layer bonding
- Figure S13. SEM characterization of electroless Cu deposition within nanochannels
- Figure S14. Control experiment for electroless Cu deposition without an Au seed layer
- Figure S15. Optimization of Au seed-layer thickness and electroless Cu deposition time
- Figure S16. XPS characterization of copper deposited inside the nanochannels
- Figure S17. Fluorescence microscopic imaging of dye-filled channels
- Figure S18. Drift-diffusion and bulk measurements of KCl
- Figure S19. Ion transport and control measurements
- Figure S20. Effect of voltage frequency on memristive loops
- Figure S21. Control measurements of electrodes

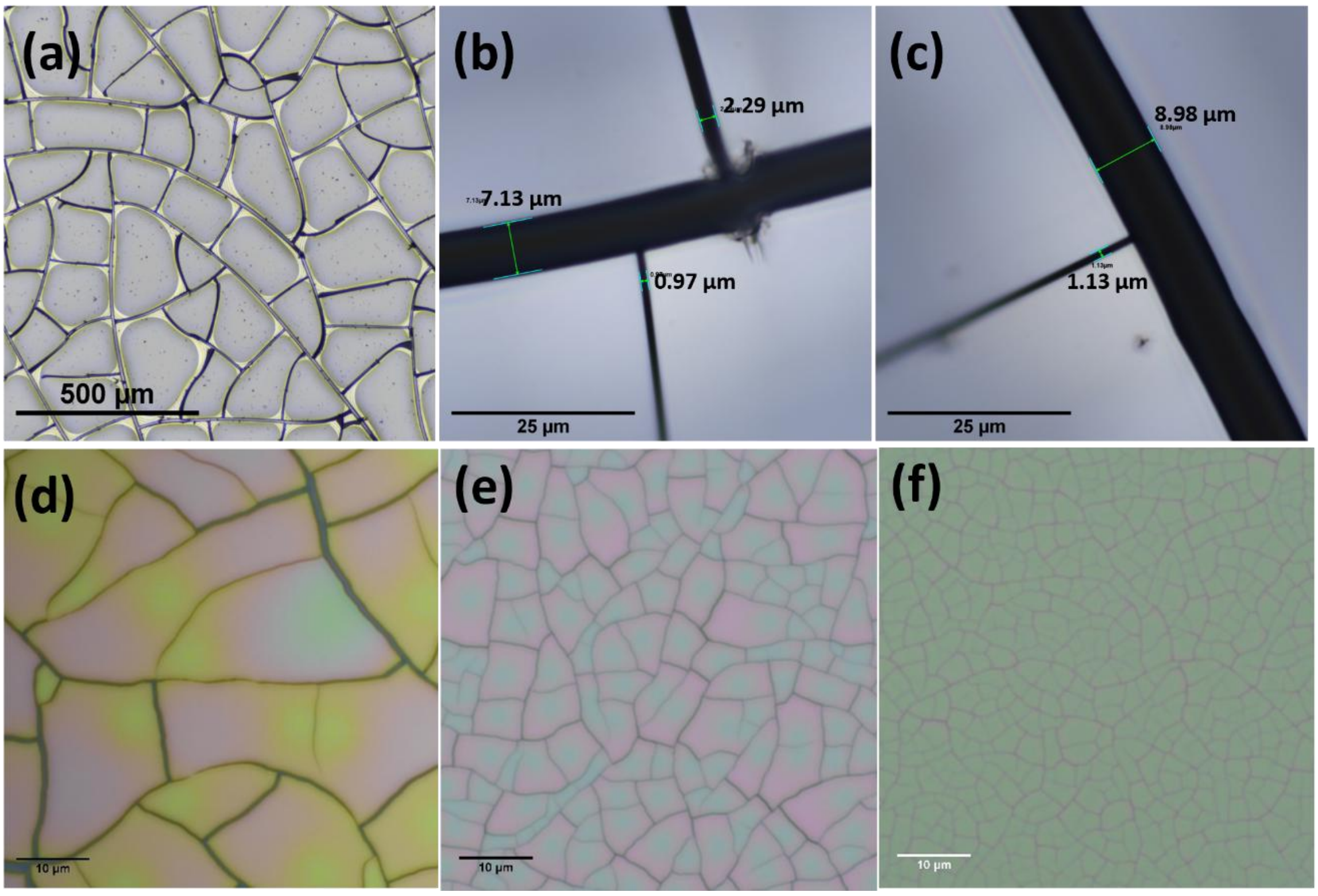


**Figure S1. Control of crack patterns.** Optical microscope images showing crack patterns generated using different precursor solutions and coating conditions. Images **(a-c)** present patterns formed by using egg white as the crackle precursor (egg white:water = 3:1), drop-coated onto a $SiO_2$ substrate. Images **(d-f)** show patterns obtained from a $SiO_2$ nanoparticle precursor deposited onto $SiO_2$ substrates at spin-coating speeds of 1000 rpm, 3000 rpm, and 5000 rpm, respectively. These results highlight the ability of the method to tune channel widths from several micrometres down to a few hundred nanometres.

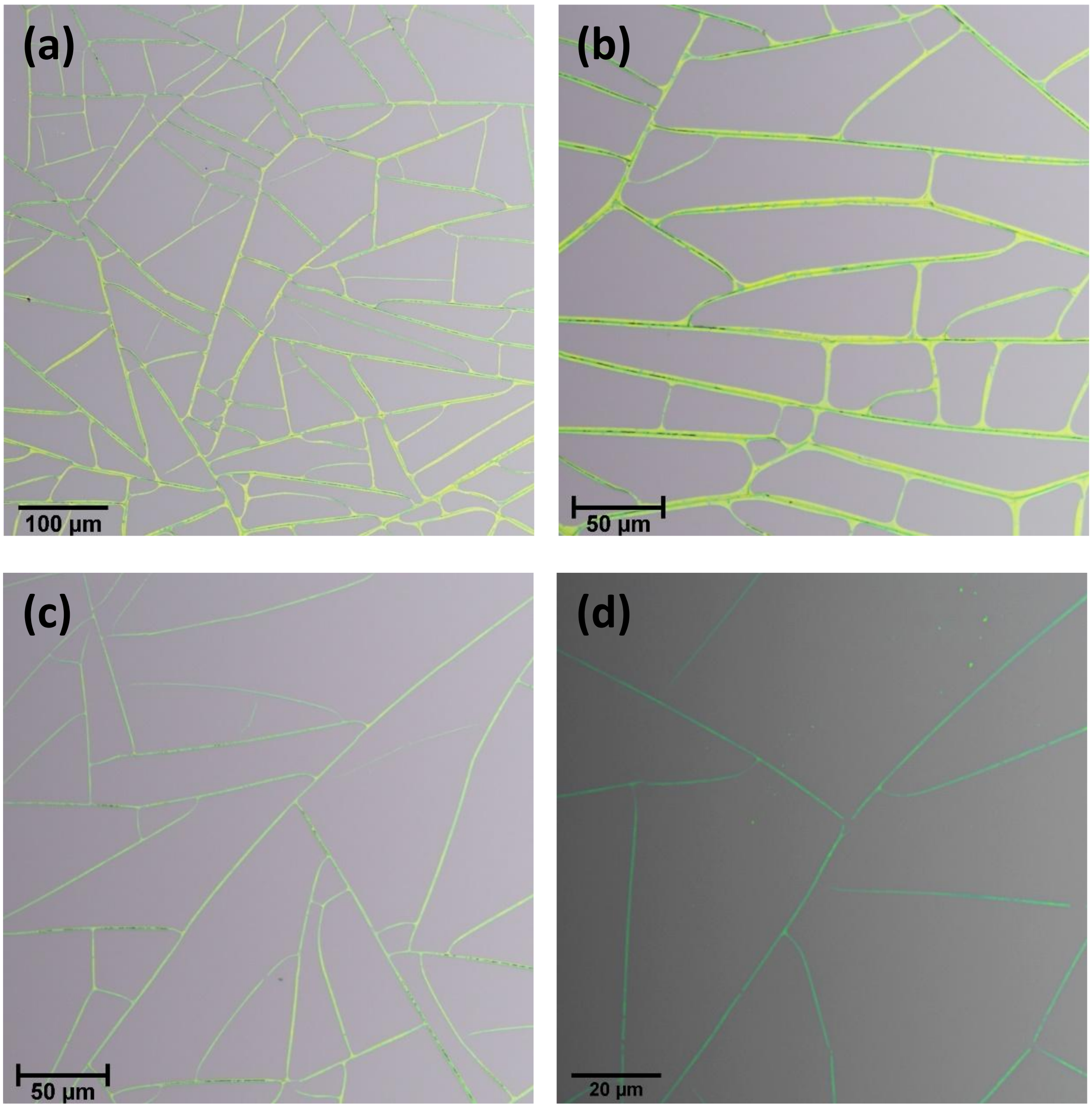


**Figure S2. Gold conductive electrode fabricated using a crackle template mask. (a-d)** Optical microscope images of gold conductive electrodes formed by thermal evaporation of gold onto a $SiO_2$ wafer, using an egg white (EW) crackle template as a shadow mask.

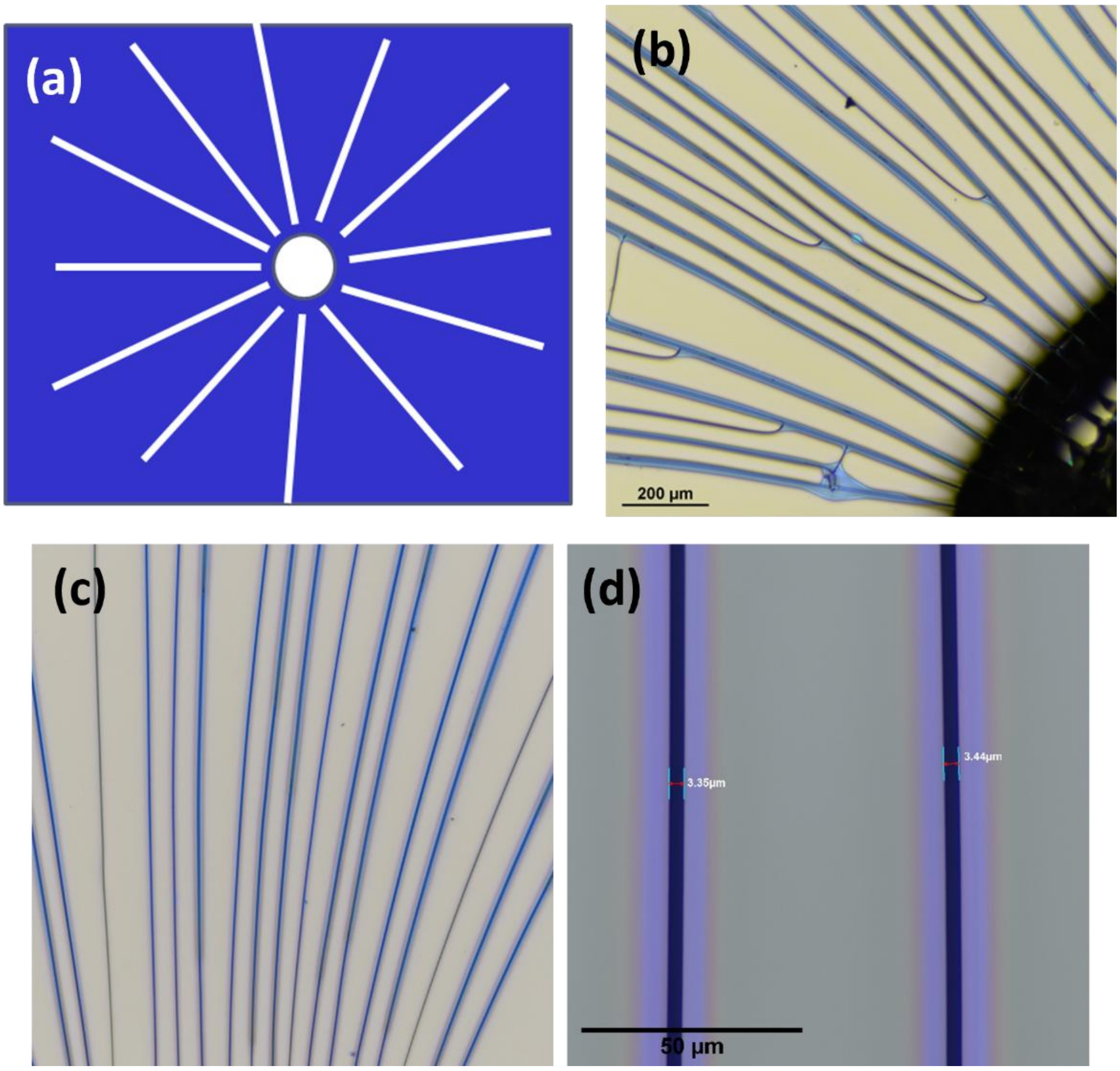


**Figure S3. Radially guided cracks.** The egg white-based crackle precursor forms radially oriented cracks upon solvent evaporation under vacuum. **(a)** Schematic illustration of the resulting radial crack pattern. **(b-d)** Optical microscope images showing the formation of cracks propagating from the centre towards the edges of the substrate.

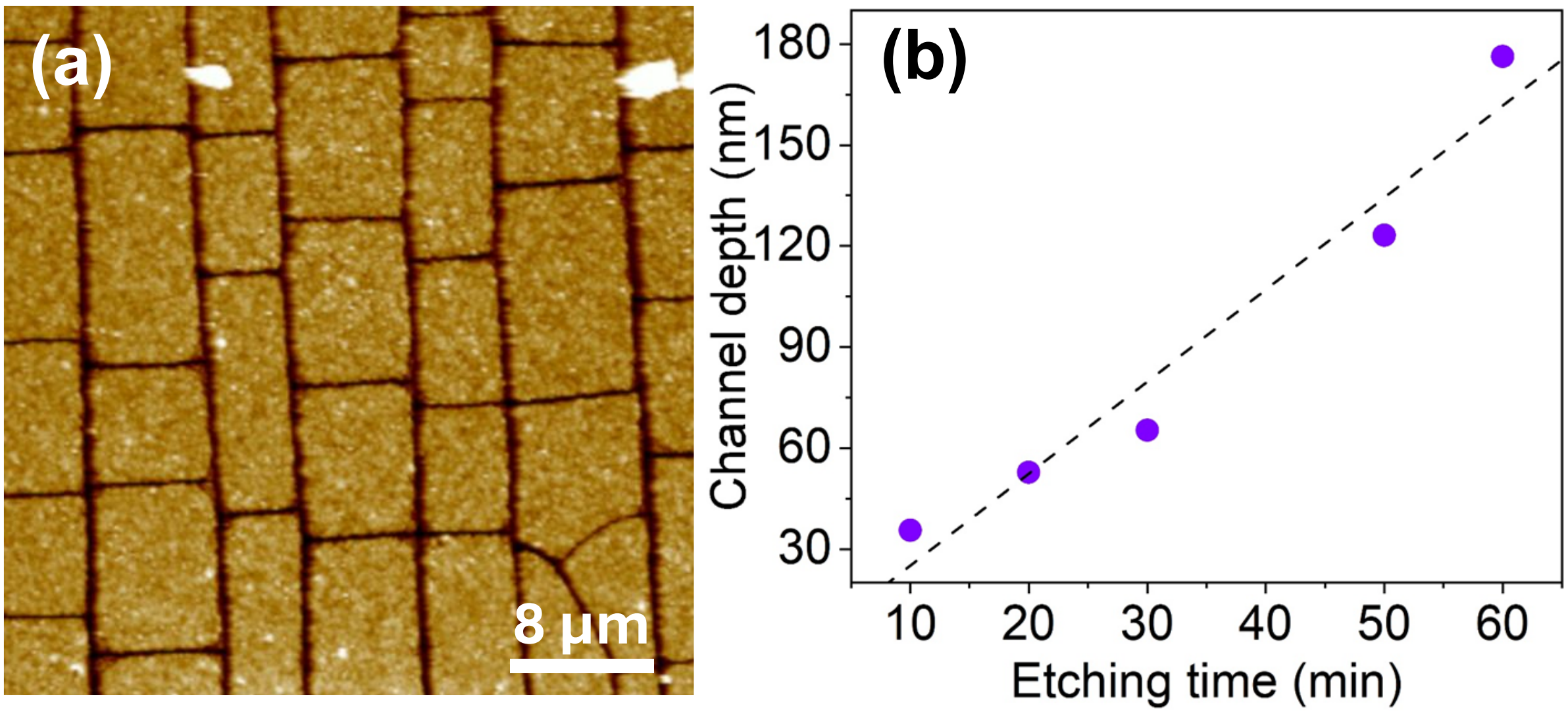


**Figure S4. Depth analysis of PET nanochannels. (a)** Atomic force microscopy (AFM) image of nanochannels formed on PET substrates. **(b)** Channel depth as a function of oxygen plasma etching time for PET substrates. The dashed line represents a linear fit, serving as a guide to the eye.

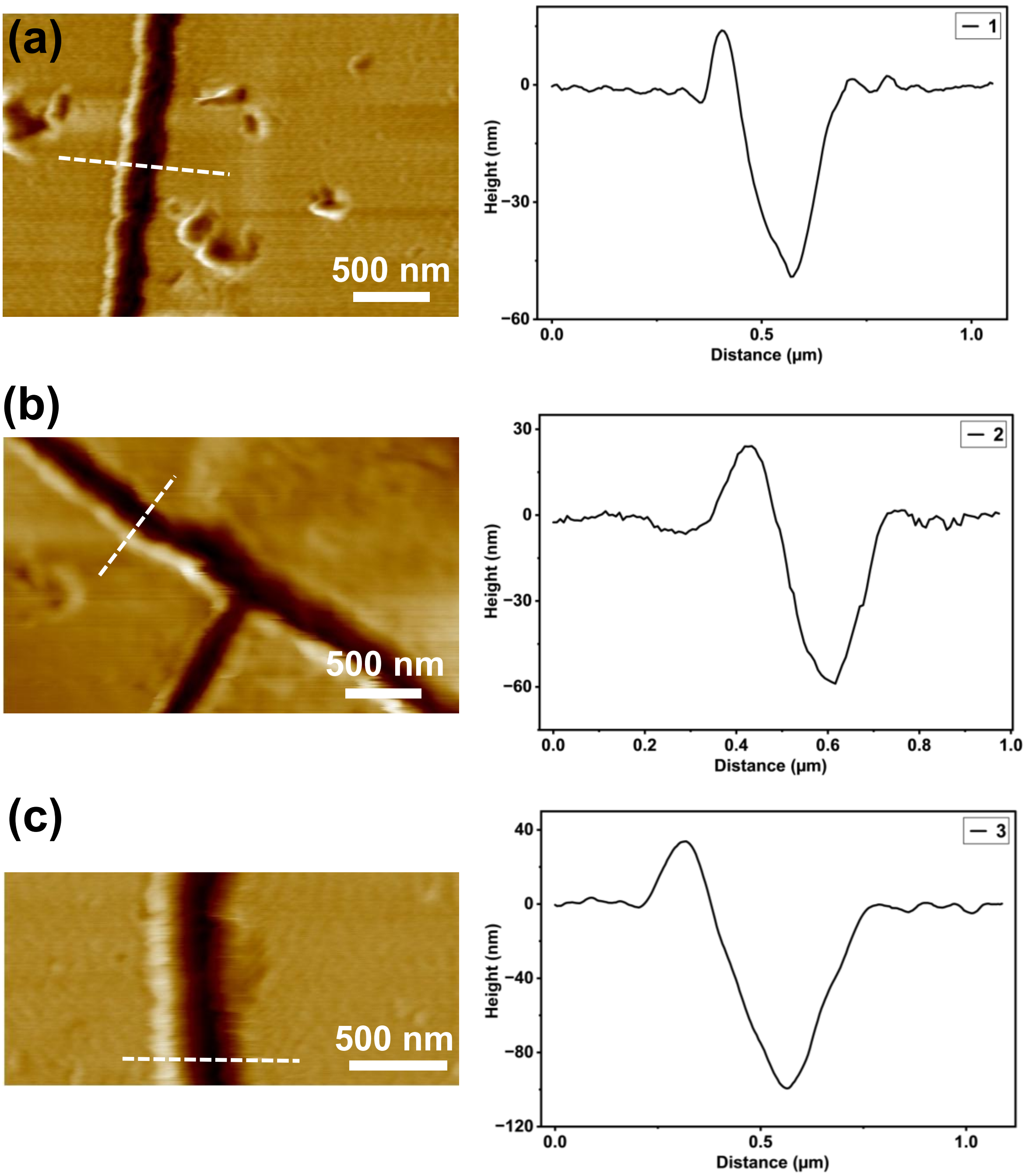


**Figure S5. AFM analysis of channel depth and width. (a-c)** AFM profiles of individual nanochannels fabricated using the crack-lithography technique, illustrating the channel depths and widths corresponding to the AFM images shown above.

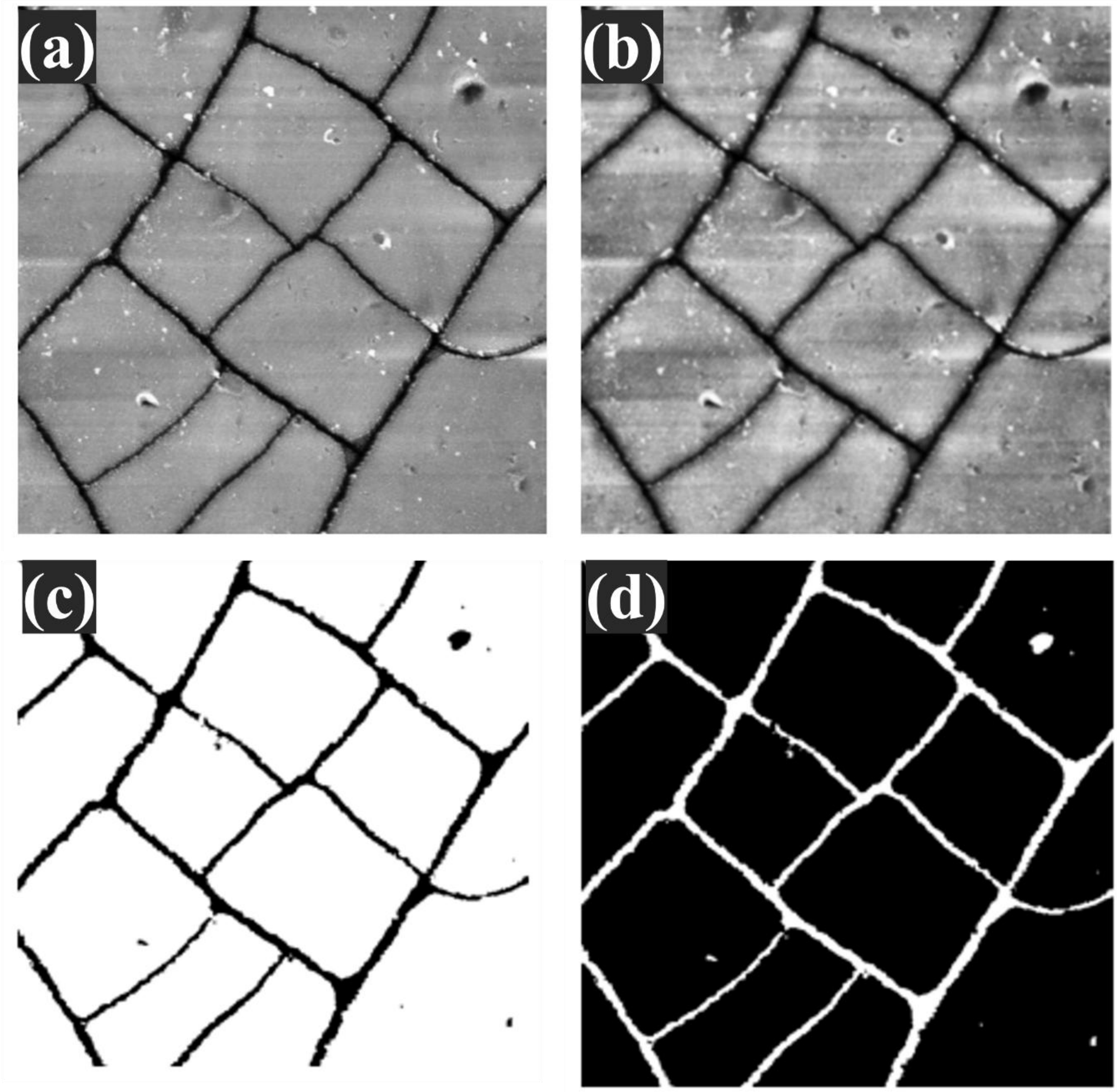


**Figure S6. Sequential image processing for width analysis of nanochannels. (a)** Original atomic force microscope image of the crack pattern. **(b)** Contrast Limited Adaptive Histogram Equalization (CLAHE) applied to enhance the image contrast followed by Gaussian blurring to reduce noise. **(c)** The image was then binarized using Otsu's thresholding. **(d)** The binarized image was inverted, and Euclidean distance transform was performed. The widths of the nanochannels were then measured by identifying the centre of the crack and the mean crack width is calculated. The Python code is available at the GitHub repository (https://github.com/tirumalarao439/Plastic-nanofluidic-devices-fabricated-using-unconventional-crackle-lithography.git).

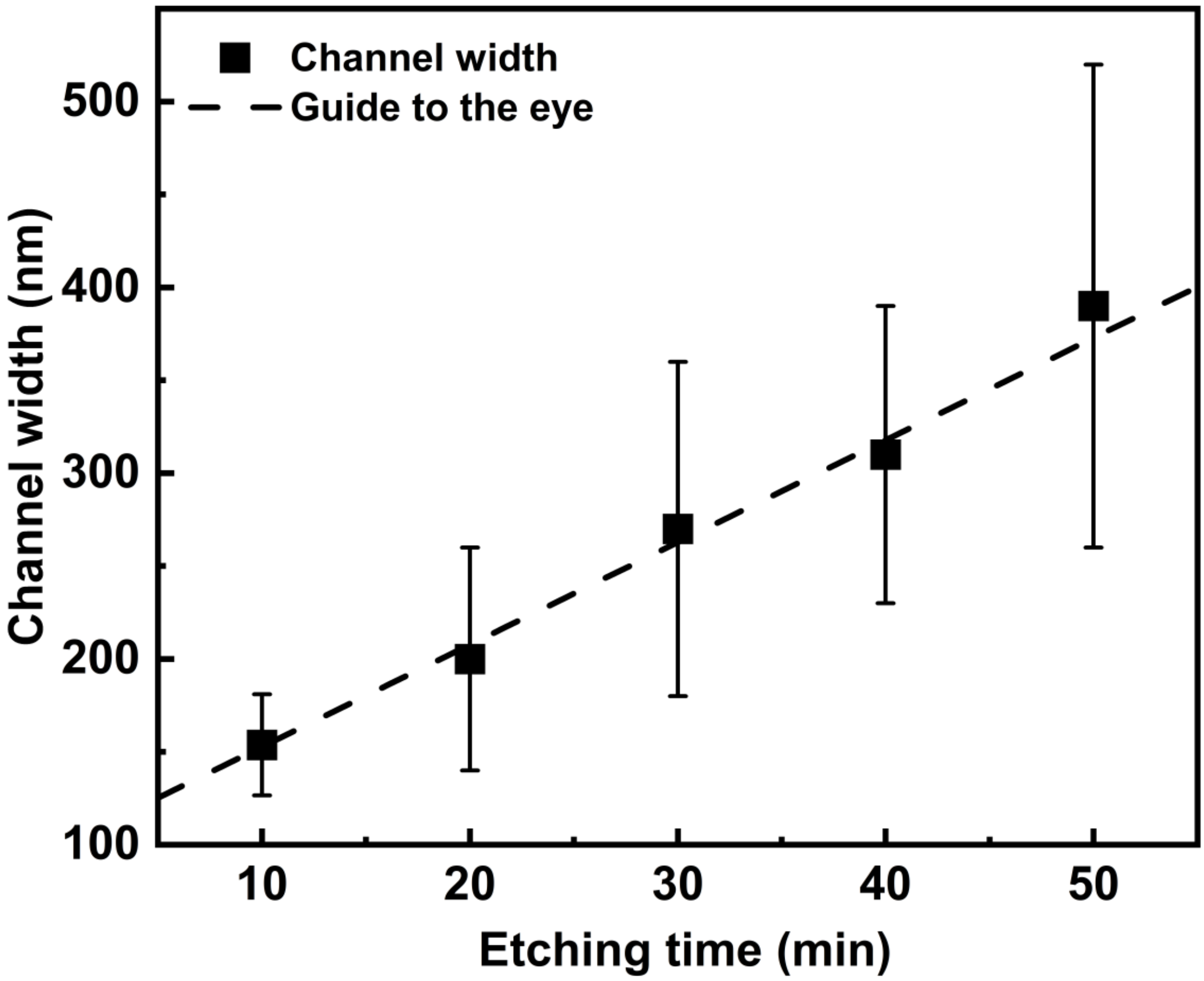


**Figure S7. Width analysis of nanochannels *via* sequential image processing.** Mean channel width as a function of etching time, quantified from AFM images using the sequential image-processing workflow described in **Fig. S6** (contrast enhancement → denoising → Otsu binarization → inversion → Euclidean distance transform with centreline detection). The analysis reveals an approximately linear increase in mean channel width with etching time, indicating controllable widening of the nanochannels through timed etching.

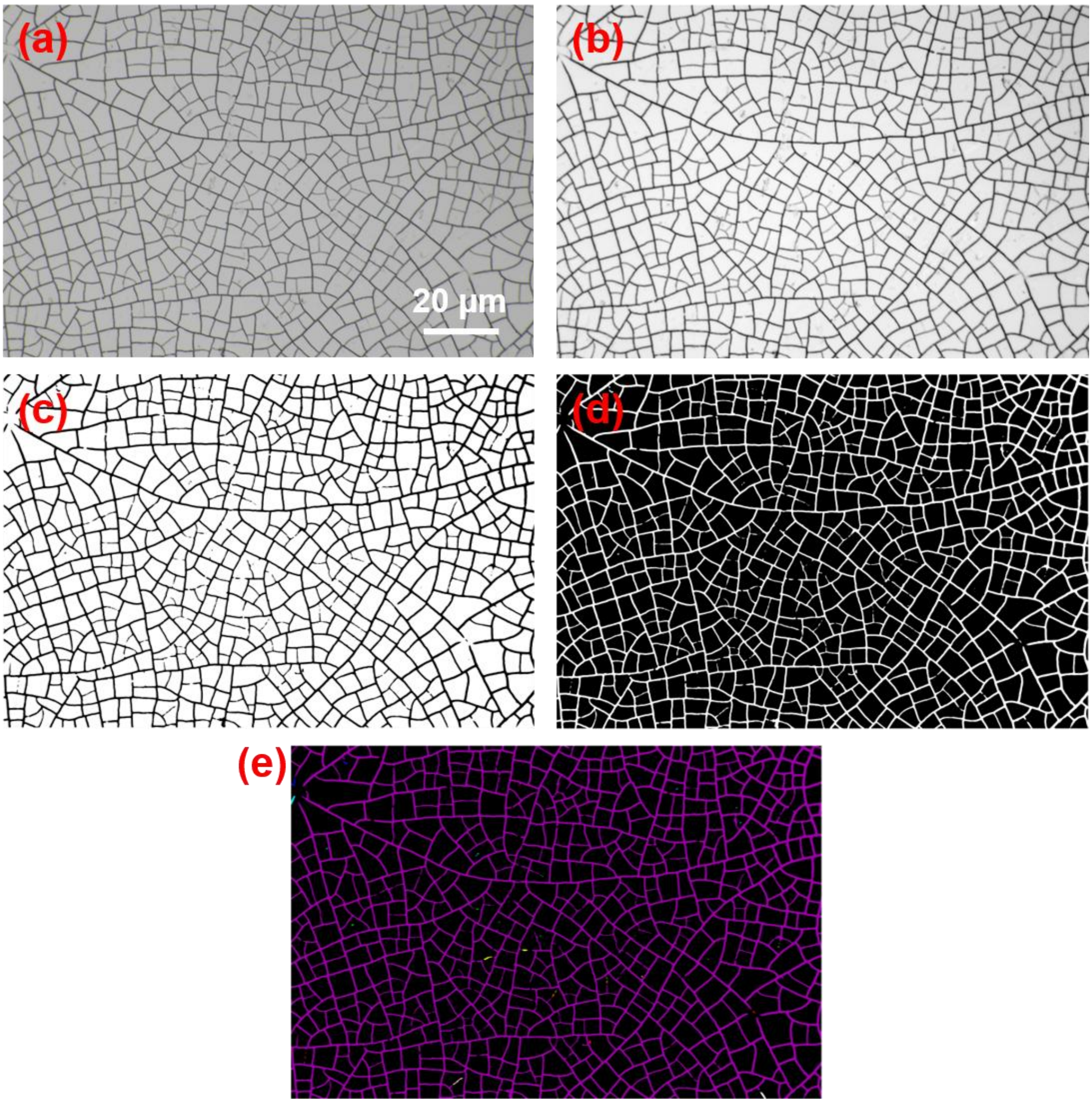


**Figure S8. Image-processing workflow used for crack-area and connectivity analysis of the crackle-patterned network**. **(a)** Original optical microscopy image of the crack network. **(b)** Grayscale image used for image processing. **(c)** Binary image obtained after Otsu thresholding to separate crack regions from the background. **(d)** Inverted crack mask in which crack regions are represented by white pixels and the background by black pixels. **(e)** Connected-component map obtained from the crack mask, with contrast enhancement applied to improve visualization of the dominant interconnected crack region. Crack coverage was calculated as the fraction of crack pixels within the analysed image area, while the largest connected component fraction was calculated as the area of the largest connected crack network divided by the total crack area. The Python code is available at the GitHub repository (https://github.com/tirumalarao439/Plastic-nanofluidic-devices-fabricated-using-unconventional-crackle-lithography.git).

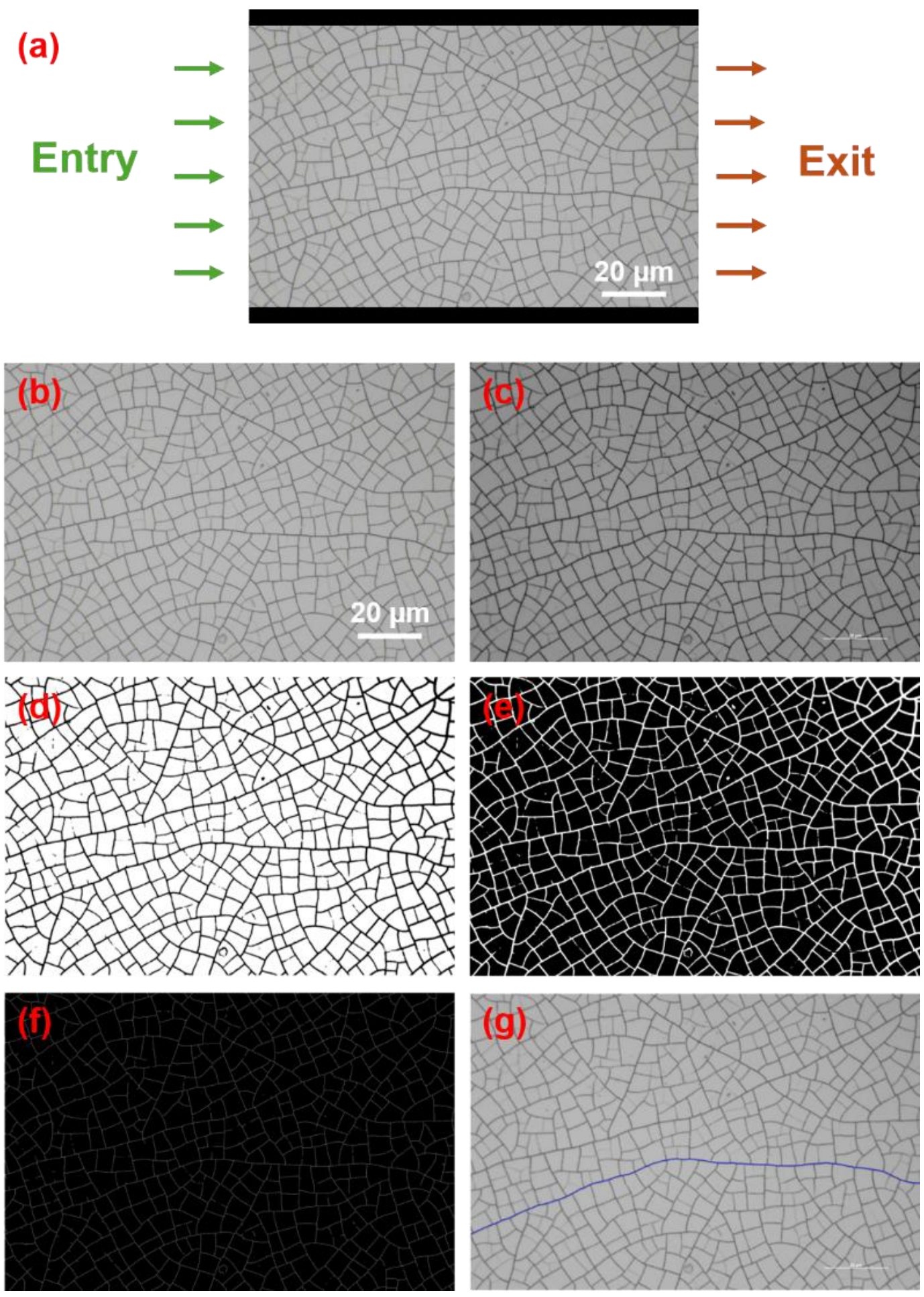


**Figure S9. Sequential image-processing workflow for quantitative crack-network transport analysis**. **(a)** Schematic illustrating the model assumption used for image analysis, where transport is allowed from the left edge (entry) to the right edge (exit), while the top and bottom boundaries were treated as blocked boundaries. **(b)** Original optical microscopy image of the crack network. **(c)** Image contrast enhanced using Contrast Limited Adaptive Histogram Equalization (CLAHE) to improve crack visibility. **(d)** Conversion of the enhanced image into a binary representation using Otsu's thresholding, followed by morphological closing to remove local discontinuities in the crack network. **(e)** Inverted binary image highlighting the crack network as white pixels on a black background. **(f)** Skeletonization of the processed image to obtain a one-pixel-wide representation of the crack-channel network. **(g)** Shortest transport pathway across the crack network extracted by applying Dijkstra's algorithm to the skeletonized image. The same graph-based framework was further used to calculate the distribution of all valid left-to-right spanning paths, enabling quantitative extraction of the average path length and mean tortuosity of the crack network. This image-processing workflow enables quantitative analysis of effective transport pathways and interconnectivity within disordered crack-patterned networks. The Python code is available at the GitHub repository (https://github.com/tirumalarao439/Plastic-nanofluidic-devices-fabricated-using-unconventional-crackle-lithography.git).

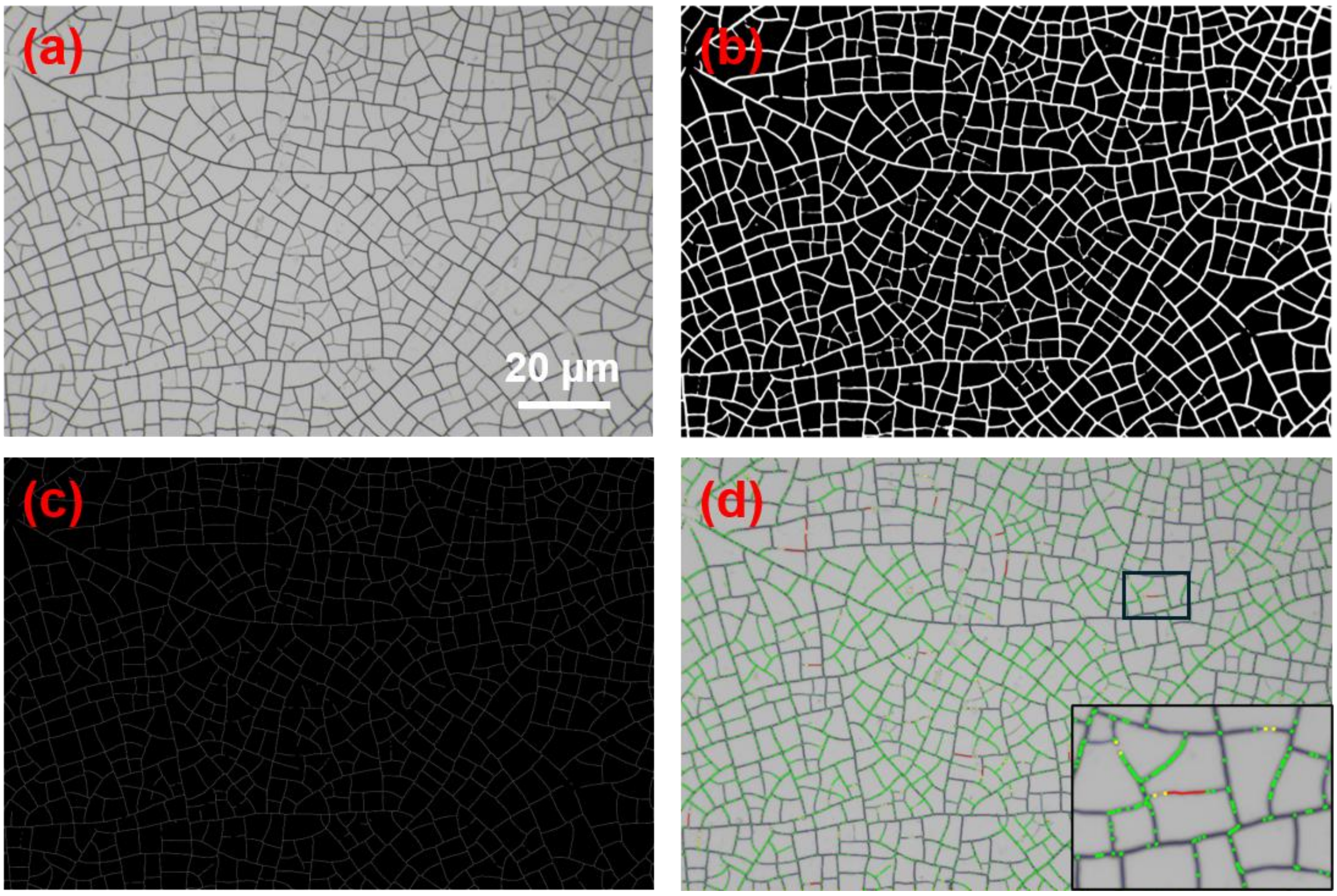


**Figure S10. Dead-end path analysis of crack-defined nanochannel networks**. **(a)** Original optical microscopy image of the crack network. **(b)** Inverted binary crack mask obtained after thresholding, where crack regions are represented as white pixels and the background as black pixels. **(c)** Skeletonized representation of the crack network used for graph-based analysis. **(d)** Overlay of the detected dead-end pathways on the original optical image, with the full skeletonized crack network shown in black, dead-end paths highlighted in red, endpoints marked in yellow, and branch points marked in green. The boxed region and zoomed-in inset show a representative dead-end pathway. Dead-end pathways were identified by tracing terminal branches from each endpoint to the nearest branch point in the skeletonized network. The dead-end pathway fraction was calculated as the cumulative length of all dead-end branches divided by the total skeletonized crack-network length.

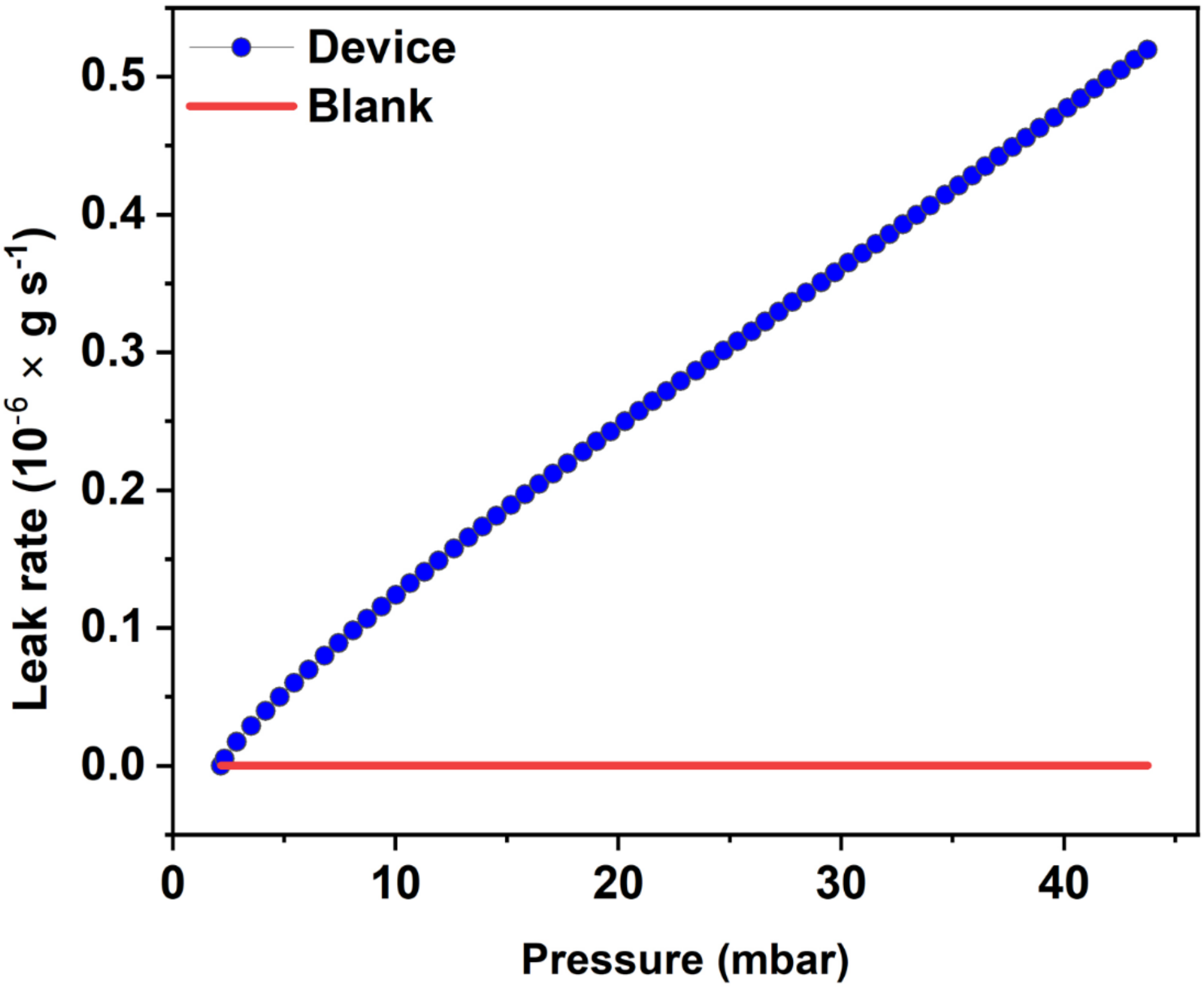


**Figure S11. Helium leak measurements.** Helium leak rate as a function of applied pressure for a blank substrate (red) and a nanochannel device (blue).

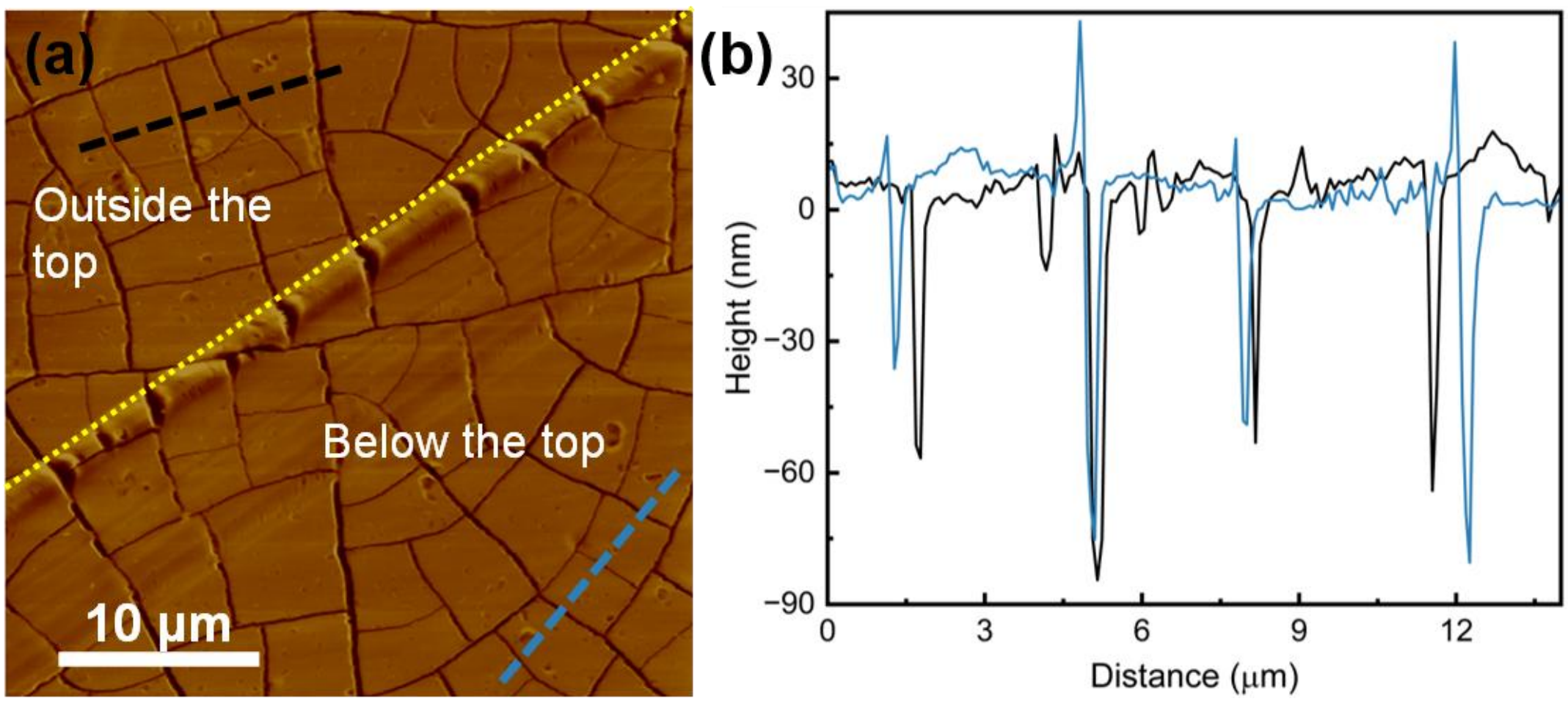


**Figure S12. Stability of nanochannels after top-layer bonding**. **(a)** AFM image acquired near the boundary between a region previously covered by the bonded top PETG and an uncovered region following careful delamination. The yellow dotted line indicates the approximate boundary of the bonded area. **(b)** Representative height profiles extracted from the regions inside (blue dashed line) and outside (black dashed line) the bonded area in panel **(a)**. The comparable channel dimensions and morphologies indicate that the bonding process does not significantly alter the nanochannel structure.

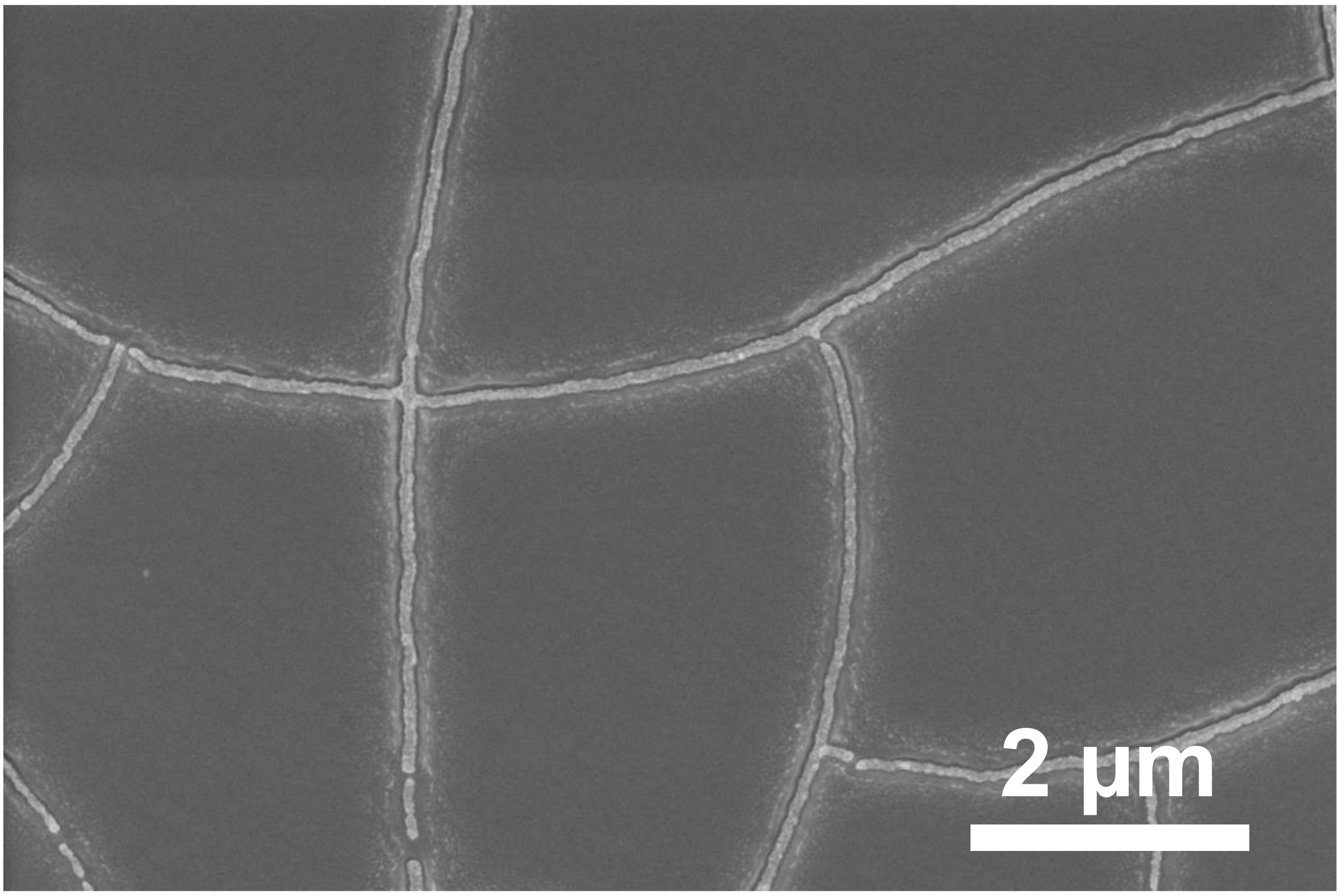


**Figure S13. SEM characterization of electroless Cu deposition within nanochannels.** Representative SEM image acquired after electroless Cu deposition. The observed metal growth along the nanochannel pathways confirms that the plating solution successfully accessed the channel network. The local extent of deposition may vary with channel width and connectivity.

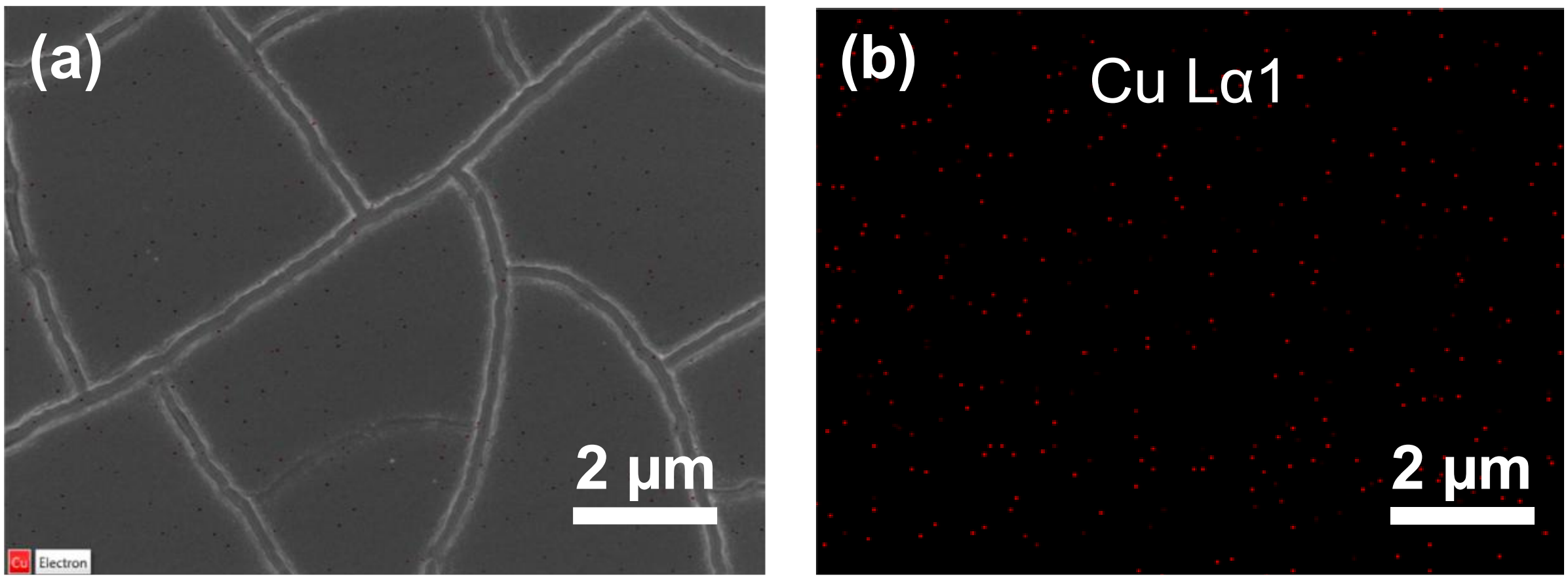


**Figure S14. Control experiment for electroless Cu deposition without an Au seed layer. (a)** SEM image of the channel region without an Au seed layer after exposure to the electroless Cu deposition solution. **(b)** Corresponding Cu EDS elemental map, showing no detectable Cu deposition along the channel paths under these conditions. This result supports the role of the Au seed layer in enabling localized Cu deposition inside the nanochannel network.

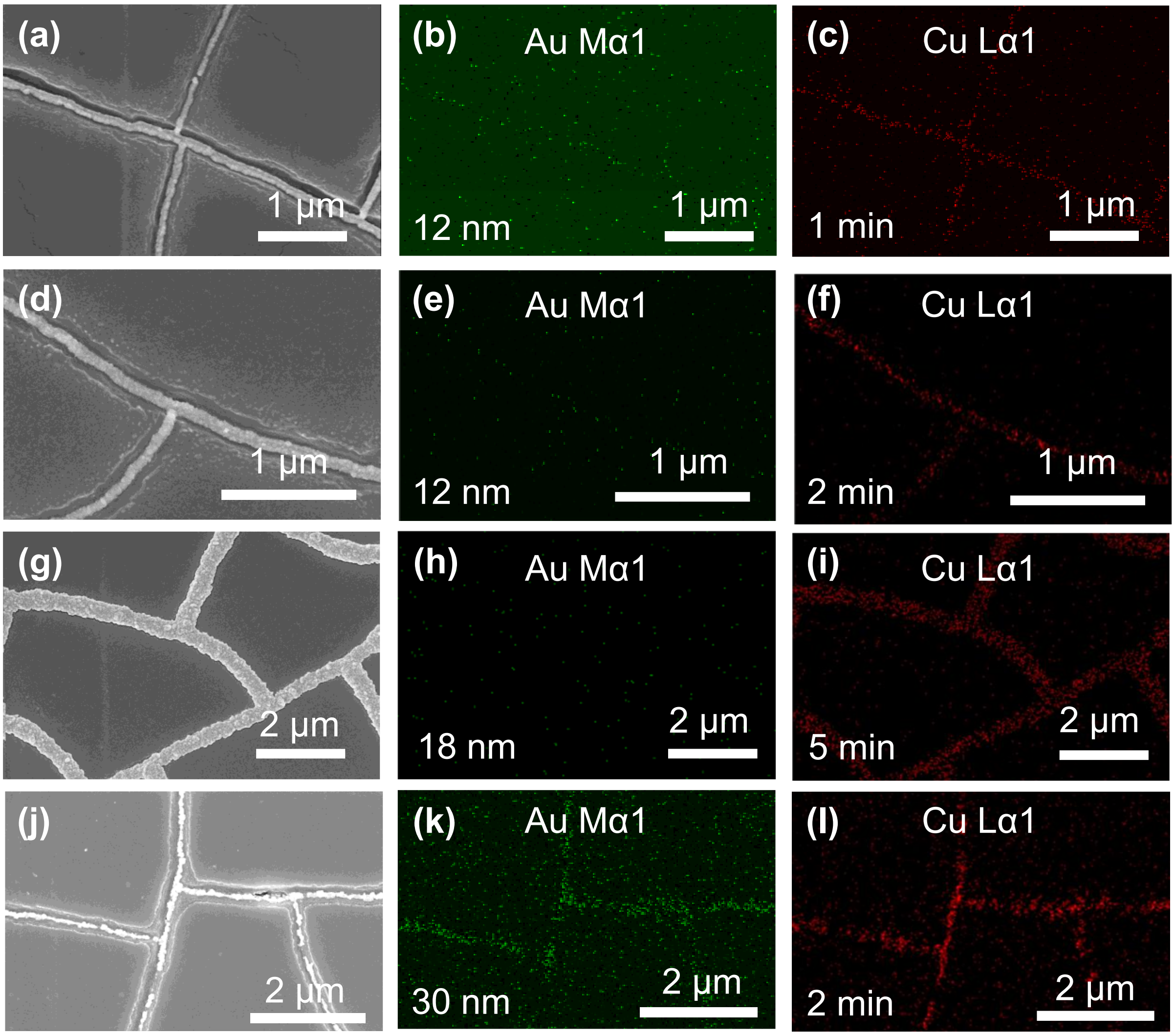


**Figure S15**. **Optimization of Au seed-layer thickness and electroless Cu deposition time.** (**a**-**c**) SEM image and corresponding Au and Cu EDS elemental maps for channels with a 12 nm Au seed layer after 1 min of electroless Cu deposition. (**d**-**f**) SEM image and EDS elemental maps for a 12 nm Au seed layer after 2 min of Cu deposition. (**g**-**i**) SEM image and EDS elemental maps for an 18 nm Au seed layer after 5 min of Cu deposition. (**j**-**l**) SEM image and EDS elemental maps for a 30 nm Au seed layer after 2 min of Cu deposition. The Au signal was not clearly detected for the 12 nm and 18 nm seed layers under the present EDS mapping conditions, whereas the 30 nm Au layer provided a detectable Au signal. For Cu deposition, 1 min resulted in a weak Cu signal, while 5 min led to excessive Cu growth. The optimized condition was therefore selected as a 30 nm Au seed layer with 2 min electroless Cu deposition, which provided clearer EDS signals for both Au and Cu along the channel paths.

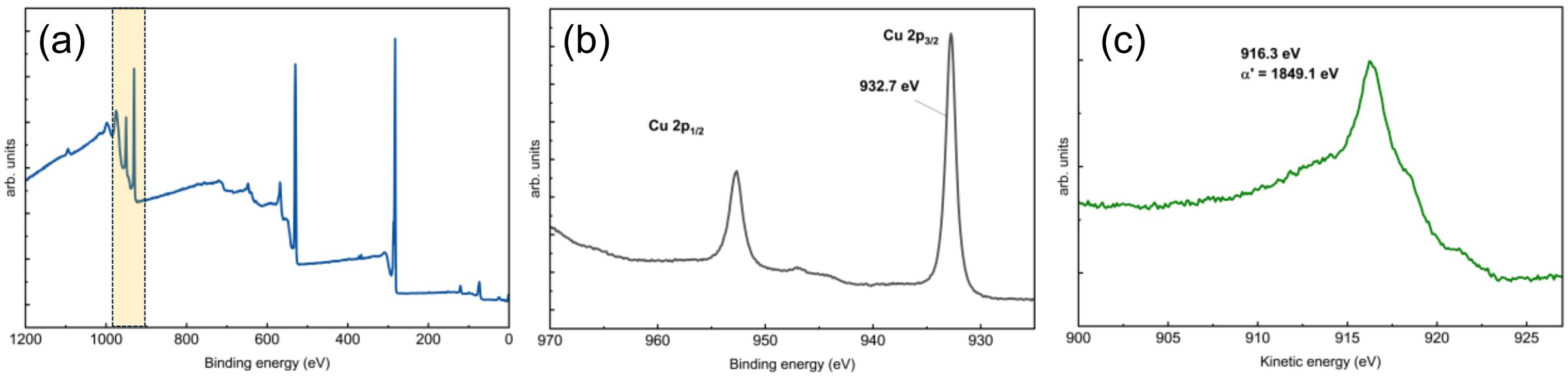


**Figure S16. XPS characterization of copper deposited inside the nanochannels**. (**a**) XPS survey spectra of the Cu-deposited nanochannel samples, confirming the presence of Cu together with the expected substrate-related signals. Binding energy scale calibration was performed using C-C in the C 1s photoelectron peak at 285 eV. (**b**) High-resolution Cu 2p spectra showing the Cu $2p_{3/2}$ and Cu $2p_{1/2}$ core-level peaks. Only weak satellite features are observed, indicating that Cu(II)-rich species such as CuO are not dominant in the deposited copper. (**c**) Cu LMM Auger spectra used to determine the modified Auger parameter, α′. The measured value of α′ = 1849.1 ± 0.2 eV is consistent with Cu(I)/$Cu_2O$, suggesting that the deposited Cu surface is mainly present as a Cu(I)-rich native oxide/passivation layer. The absence of strong Cu(II)-related satellite features further supports that CuO/$Cu(OH)_2$ is not the dominant surface species.

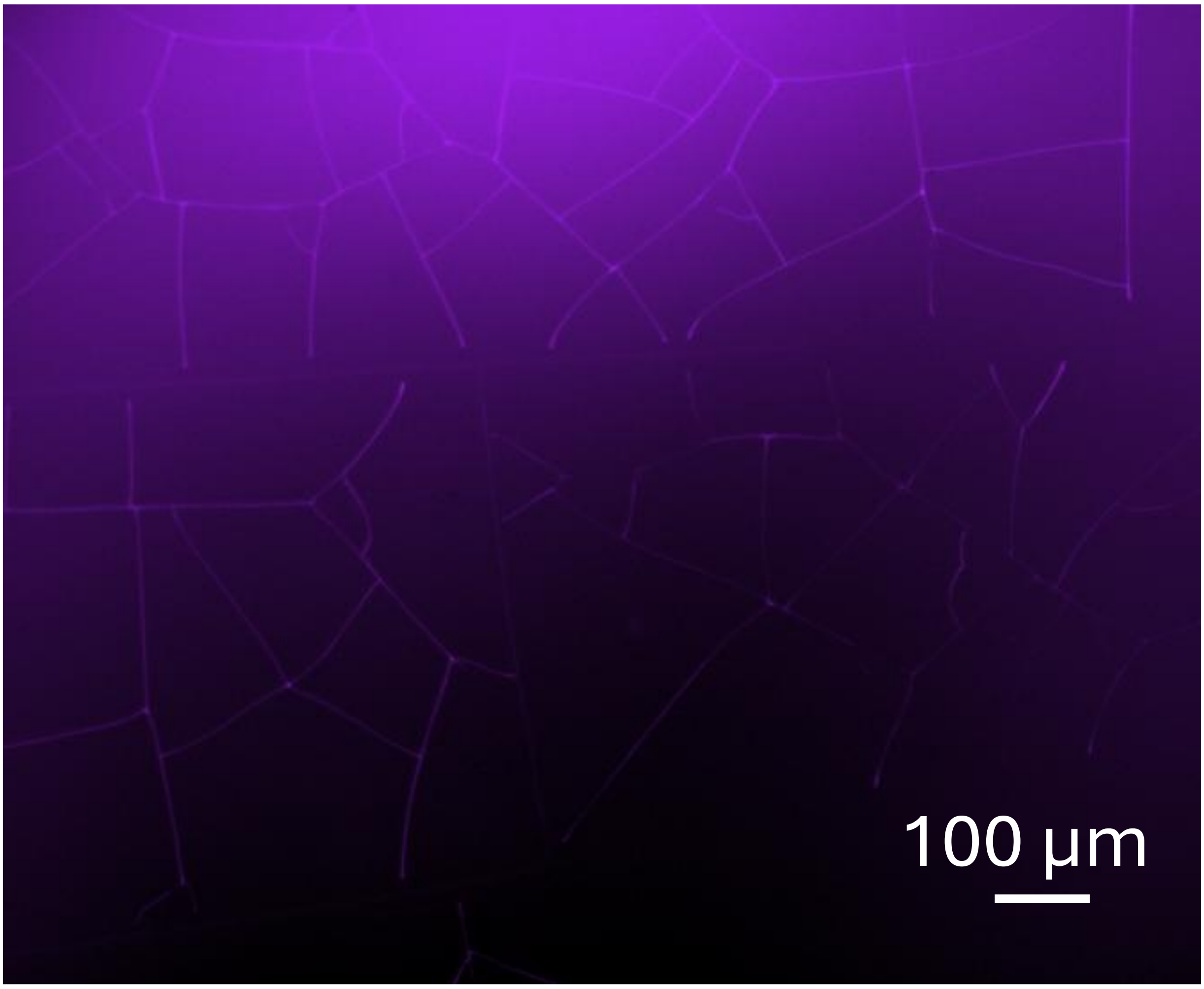


**Figure S17. Fluorescence microscopic imaging of dye-filled channels.** Rhodamine 6G dye was introduced into the crack-patterned PETG nanochannels (etched for 30 minutes) and illuminated with UV light under a fluorescence microscope. Channels imaged away from the hole (entry point) region. It validates the consistent infiltration of dye into the nanochannel network.

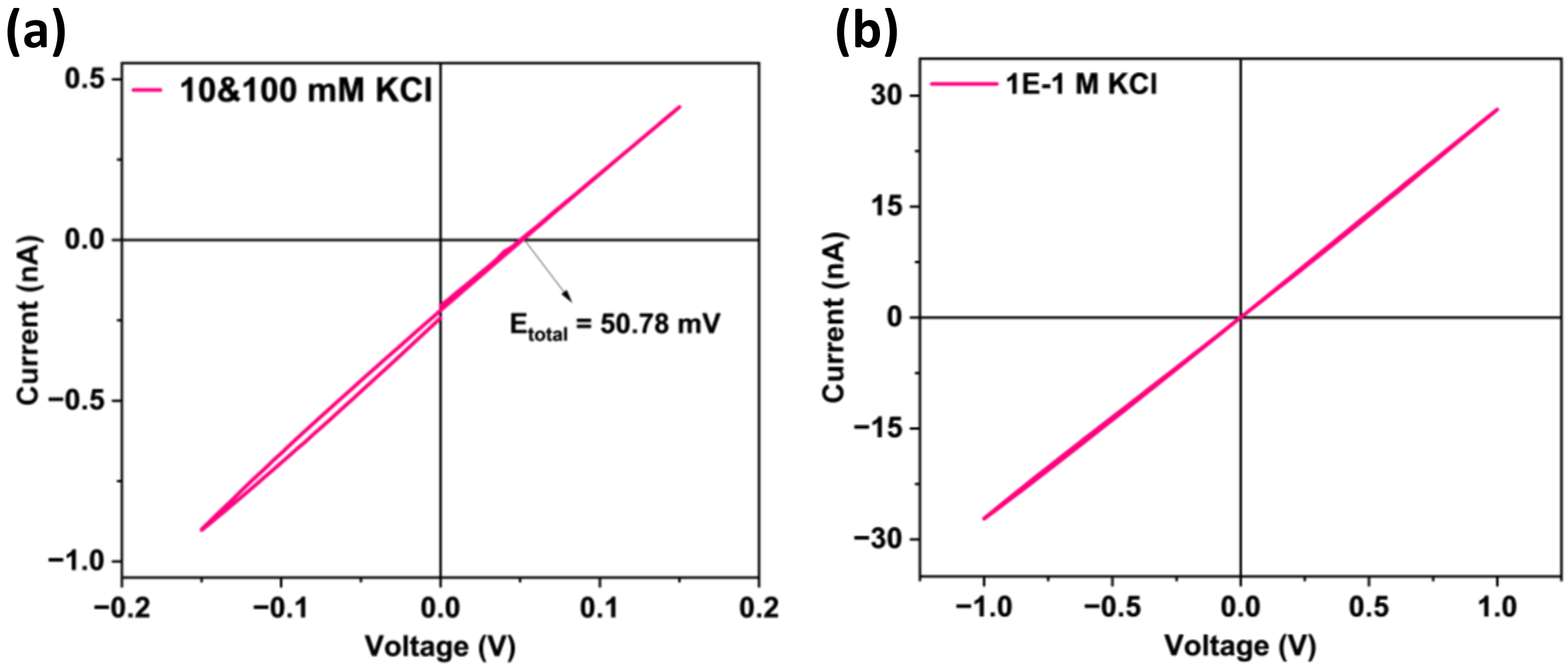


**Figure S18. Drift-diffusion and bulk measurements of KCl. (a)** Current–voltage (*I*-*V*) characteristics of drift-diffusion experiments recorded through a 10 min-etched PETG nanochannels device using standard Ag/AgCl electrodes with asymmetric KCl concentrations in the feed and permeate reservoirs 100 mM and 10 mM, respectively. The redox potential of the Ag/AgCl electrodes (≈55 mV) defines the zero-current potential of the device, yielding effective membrane potential of 5 mV, which corresponds to bulk-dominated transport. **(b)** *I*-*V* characteristics of symmetric 0.1 M KCl exhibiting bulk-like ionic transport behaviour of PETG nanochannel device.

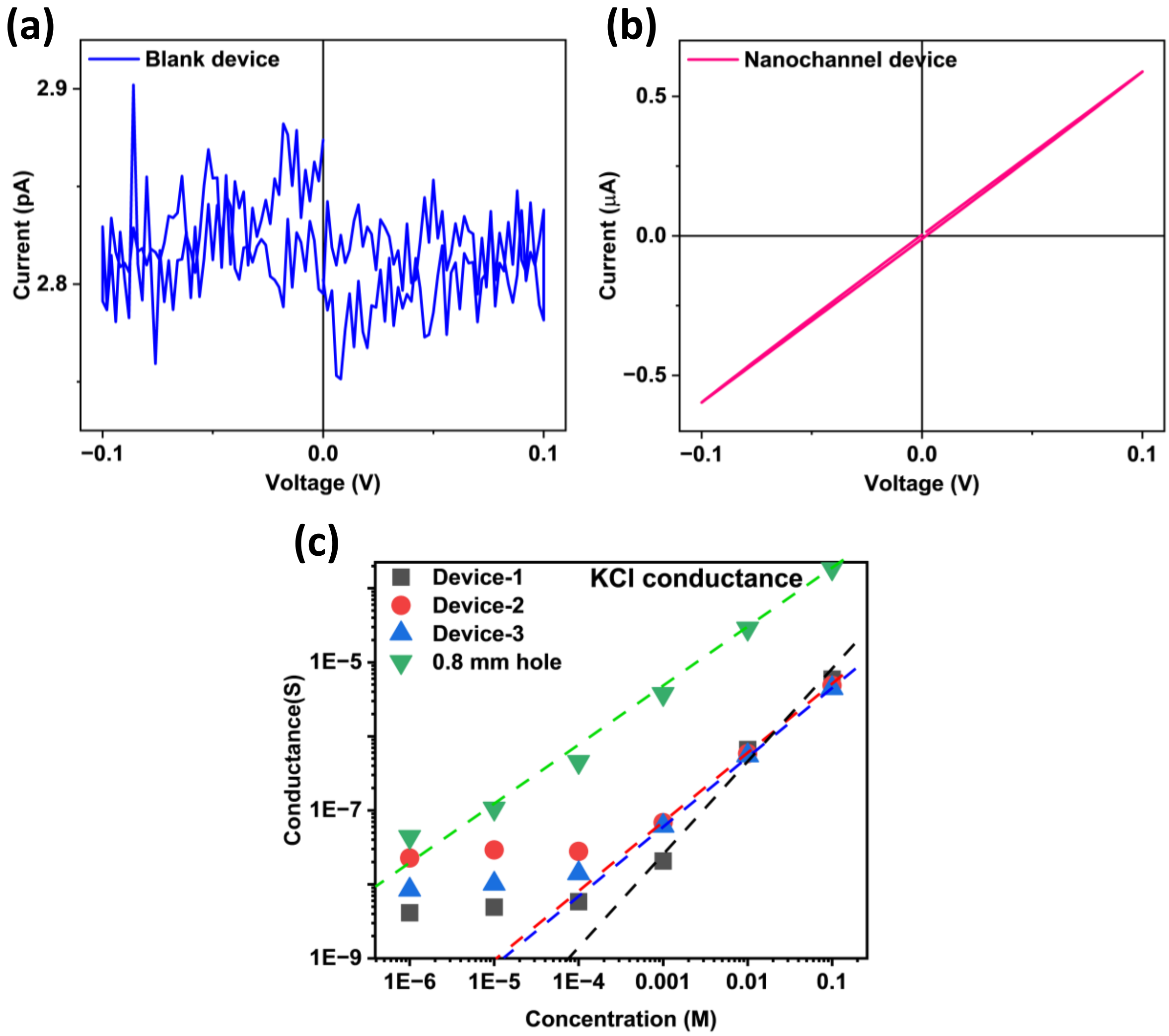


**Figure S19. Ion transport and control measurements.** Current-voltage characteristics of 0.1 M KCl for **(a)** a blank device bonded without etched channels on PETG and **(b)** a nanochannel device etched for 10 minutes. The blank device shows no measurable ionic conduction, with current values in the picoampere range corresponding to instrument noise, whereas the nanochannel device exhibits measurable ionic current in the sub-microampere range under identical conditions. These control measurements confirm that ionic transport arises solely from the presence of the etched nanochannels. **(c)** Conductance-concentration characteristics of KCl electrolyte solutions measured across devices exhibiting nanoconfinement within the fabricated nanochannels when compared to a 0.8 mm hole in the PETG substrate. The conductance values of all devices remain comparable in the bulk regime, with slight variations observed in the surface charge-dominated region, likely arising from multiple parallel conduction paths within the nanochannel network.

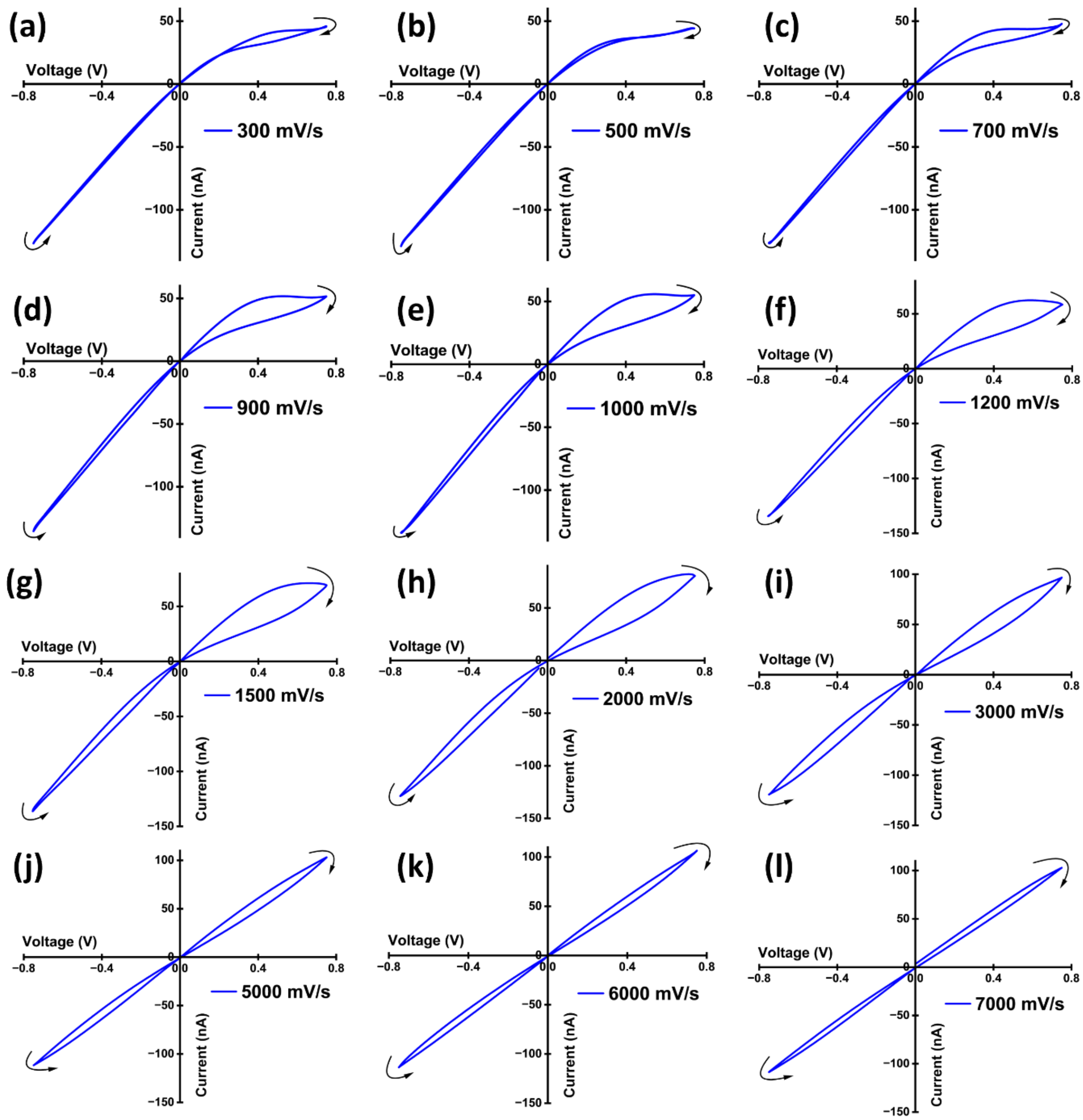


**Figure S20. Effect of voltage frequency on memristive loops. (a-l)** Current–voltage characteristics of 300 mM $AlCl_3$ solutions recorded at scan rates ranging from 300 mV $s^{-1}$ to 7000 mV $s^{-1}$.

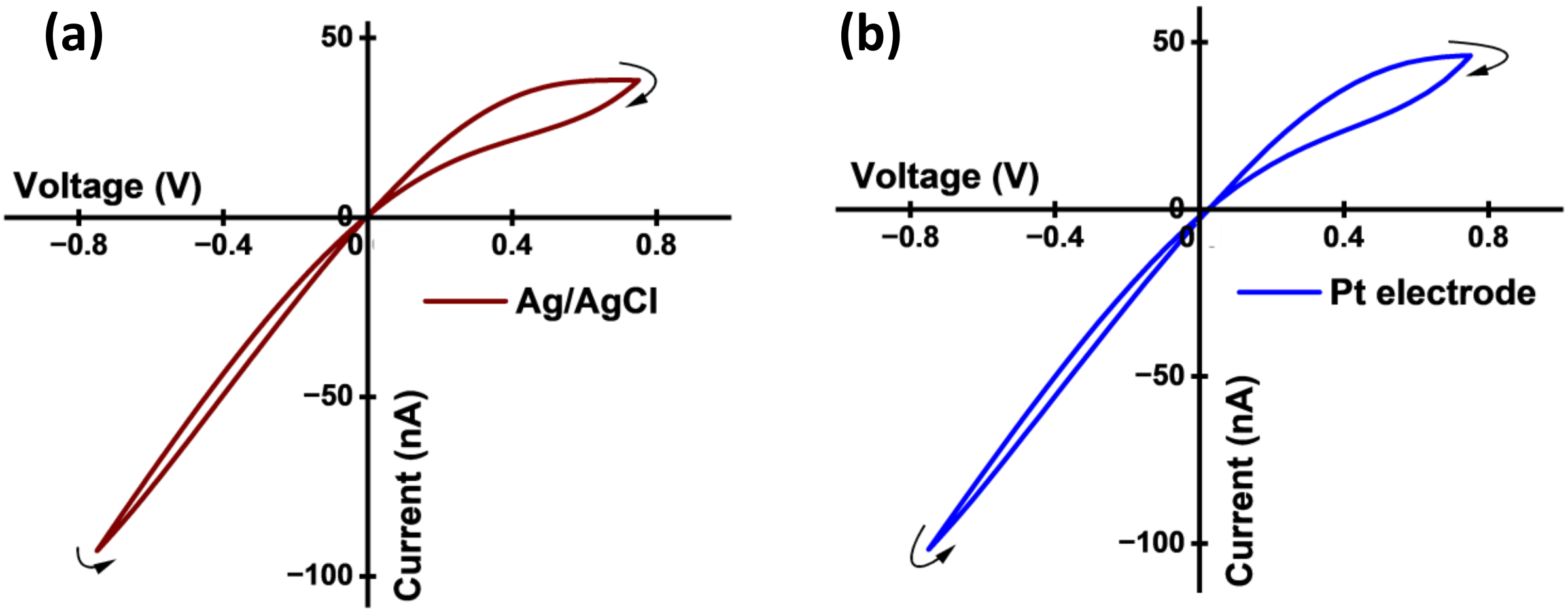


**Figure S21. Control measurements of electrodes.** The reproducibility of memristive loops was verified using **(a)** Ag/AgCl and **(b)** Pt electrodes.
